# VGF and the VGF-derived peptide AQEE30 stimulate osteoblastic bone formation through the C3a receptor

**DOI:** 10.1038/s12276-025-01419-y

**Published:** 2025-03-13

**Authors:** Sung-Ah Moon, Jin-Man Kim, Young-Sun Lee, Han Jin Cho, Young Jin Choi, Jong Hyuk Yoon, Dayea Kim, Xiangguo Che, Xian Jin, In-Jeoung Baek, Seung Hun Lee, Je-Young Choi, Jung-Min Koh

**Affiliations:** 1 Department of Medical Science, AMIST, Asan Medical Center, University of Ulsan College of Medicine, Seoul, Republic of Korea; 2 Asan Institute for Life Sciences, Asan Medical Center, Seoul, Republic of Korea; 3 Department of Life Sciences, Pohang University of Science and Technology (POSTECH), Pohang, Republic of Korea; 4 Neurodegenerative Disease Research Group, Korea Brain Research Institute, Daegu, Republic of Korea; 5 New Drug Development Center, Daegu-Gyeongbuk Medical Innovation Foundation (K-MEDI hub), Daegu, Republic of Korea; 6 Department of Biochemistry and Cell Biology, Cell and Matrix Research Institute, Korea Mouse Phenotyping Center, School of Medicine, Kyungpook National University, Daegu, Republic of Korea; 7 Department of Cell and Genetic Engineering, Asan Medical Center, University of Ulsan College of Medicine, Seoul, Republic of Korea; 8 Division of Endocrinology and Metabolism, Asan Medical Center, University of Ulsan College of Medicine, Seoul, Republic of Korea

**Keywords:** Bone development, Cell proliferation, Differentiation, Proteomic analysis

## Abstract

New therapeutic targets, especially those that stimulate bone formation in cortical bone, are needed to overcome the limitations of current antiosteoporotic drugs. We previously demonstrated that factors secreted from megakaryocytes (MKs) promote bone formation. Here we conducted a proteomic analysis to identify a novel bone-forming factor from MK secretions. We revealed that *Vgf*, a nerve growth factor-responsive gene, and its derived active peptide AQEE30 in MK-conditioned medium play important roles in osteoblast proliferation and in vitro bone formation. In both *Vgf*-deficient male and female mice, the cortical bone mass was significantly decreased due to reductions in osteoblast number and bone formation activity. AQEE30 stimulated intracellular cyclic adenosine monophosphate (cAMP) levels and protein kinase A (PKA) activity in osteoblasts, whereas an adenylyl cyclase inhibitor blocked AQEE30-stimulated osteoblast proliferation and in vitro bone formation. Complement C3a receptor-1 (C3AR1) was expressed and interacted with AQEE30 in osteoblasts, and C3AR1 inhibition blocked all AQEE30-induced changes, including stimulated proliferation, bone formation and cAMP production, in osteoblasts. Injecting mini-PEGylated AQEE30 into calvaria increased the number of osteocalcin-positive cells and new bone formation. In conclusion, this study reveals a novel role of VGF in bone formation, particularly in cortical bone, and shows that AQEE30, a VGF-derived peptide, mediates this role by activating cAMP–PKA signaling via the C3AR1 receptor in osteoblasts.

## Novel factor AQEE30 enhances osteoblast activity

Osteoporosis is a common bone disease that weakens bones and increases fracture risk. Current treatments have limitations, especially in preventing nonvertebral fractures. Researchers focused on a protein called VGF, which is known for its role in the brain but not in bone health. The study used mice and cell cultures to investigate VGF’s effects on bones. They found that VGF and its peptide AQEE30 promote bone formation by stimulating osteoblasts. They used mass spectrometry to discover that VGF is important for bone health. Mice lacking VGF had weaker bones, especially in the cortical (outer) layer. The study showed that AQEE30 works by increasing cAMP levels, a molecule that helps cells communicate and grow. This discovery suggests that targeting VGF and AQEE30 could lead to new treatments for osteoporosis.

This summary was initially drafted using artificial intelligence, then revised and fact-checked by the author.

## Introduction

Osteoporosis, characterized by low bone mass and microarchitectural alterations, is the most prevalent metabolic bone disease and increases the risk of fracture^1,2^. Continuous, active bone remodeling occurs throughout life and involves osteoclast-mediated bone resorption and osteoblast-mediated bone formation^3^. Active bone resorption–formation coupling by osteoclasts and osteoblasts facilitates the maintenance of adequate bone mass^4^, whereas excess bone resorption results in low bone mass^5^. Bone resorption inhibitors and bone formation promoters can alleviate osteoporosis^6^.

Anti-osteoporotic drugs have limitations in the clinic because of their poor long-term therapeutic efficacy and nonvertebral fracture-preventive effects^7–9^. First, the limited long-term efficacy of antiresorptive agents is associated with mechanisms of bone resorption–formation coupling itself, in which the suppression of bone formation appears after decreased bone resorption^7–9^. By contrast, anabolic agents potentially have limitations in terms of the characteristics of their therapeutic targets. Parathormone-based drugs stimulate bone formation and receptor activation of the NF-κB ligand RANKL, which increases bone resorption^10^. Long-term anti-sclerostin antibody treatment becomes increasingly ineffective in promoting bone formation, possibly by suppressing Wnt-system-related compensatory mechanisms^9,11^. Thus, novel bone-formation-targeting agents, instead of bone resorption inhibitors, are necessary to overcome the limitations in the long-term efficacy of current osteoporosis therapeutic agents.

Second, another drawback is the limited nonvertebral fracture-preventive effect of the available agents. Even though most osteoporotic fractures are nonvertebral, therapeutic agents significantly reduce the risk of vertebral fracture but only slightly reduce the risk of nonvertebral fracture^12^. The main reason may be that nonvertebral bone is composed mainly of cortical bone. Moreover, these drugs primarily increase trabecular bone strength and have a less pronounced effect on cortical bone strength. Thus, new therapeutic targets to increase cortical bone strength should be identified. However, the cellular and molecular mechanisms that specifically regulate cortical bone still need to be determined.

We previously reported that conditioned medium (CM) of megakaryocytes (MKs) promoted osteoblast proliferation and increased in vivo and in vitro bone formation^13^. Here, we performed mass-spectrometry-based proteomic analysis to identify a novel bone-forming factor. We observed increased VGF expression in MK-CM and a significant decrease in cortical bone size in mice lacking *Vgf*. The VGF protein is secreted in the brain, neurons and several nervous systems^14,15^ and is cleaved proteolytically by prohormone convertases into various active peptides in different cells and tissues^16^. However, to our knowledge, there are no reports about the roles of the VGF protein and its derived peptides in bone metabolism. Here, we report that VGF and its derived active peptide AQEE30 play important roles in bone formation both in vivo and in vitro.

## Materials and methods

### Animal care

Five-week-old male C57BL/6N mice were purchased from Orient Bio. All the mice were maintained under specific-pathogen-free conditions on a 12-h light‒dark cycle at the animal facility of the Asan Institute for Life Sciences (Seoul, Korea). All the mice were provided ad libitum access to rodent chow and water. Euthanasia was performed with an intraperitoneal injection of 40 mg/kg Zoletil 50 (Virbac) and 5.6 mg/kg Rompun (Bayer Korea). No specific criteria were applied for inclusion or exclusion in the animal studies. All animal care and experimental procedures were approved by the Institutional Animal Care and Use Committee of the Asan Institute for Life Sciences (approval no. 2018-12-230).

### Cell culture and fractionated secretomics

Murine osteoblastic MC3T3-E1 and human leukemic K562 cells were purchased from the American Type Culture Collection and cultured in α-MEM and RPMI 1640 medium, both supplemented with 10% fetal bovine serum (FBS; Gibco), respectively. K562 cells (3 × 10^5^ per well in a six-well culture plate) were incubated in culture medium with or without 1 nM phorbol 12-myristate 13-acetate (PMA; Sigma-Aldrich) for 3 days to collect CM from pre-MK and MK-like cells. After the media were changed to phenol red-free α-MEM without PMA or FBS, the cells were incubated for 24 h. CM of pre-MK and MK-like cells was collected. The CM was centrifuged at 3000 rpm for 10 min (Combi-514R, Hanil Science Industry). To eliminate contaminants, the resulting supernatants were filtered through centrifugal filter units (Millipore). The filtered CM was separated into 96 fractions by reversed-phase C18 (Vydac 218TP5215, 150 mm × 2.1 mm) chromatography. Then, 500 μl of each fraction was dried and used in a cell viability assay. After the cell viability assay, the fractions were selected. For liquid chromatography–tandem mass spectrometry analysis, 100 μg of protein from each fraction was reduced in 10 mM dithiothreitol at 56 °C for 20 min and alkylated by incubation in 100 mM iodoacetamide at room temperature in the dark for 15 min. Trypsin digestion was performed for 24 h at 37 °C, after which the mixture was dried. Tryptic digests were analyzed using an LTQ Orbitrap Velos (Thermo) mass spectrometer equipped with a nano-HPLC system. Peptide identification was performed using Proteome Discoverer Thermo software with the human UniProt database. Instead of primary murine MK cells, K562-derived MK-like cells were used for secretomics to prevent excessive euthanasia of mice, exclude contamination by other cells and search for human factors. We validated the use of MK-like cells in this study as previously described^13^.

### Purification and differentiation of primary murine MK cells

For MK differentiation, the fetal livers of 15.5-day gestational (E15.5) C57BL6 mice were obtained as described previously^17^. Collected cells were cultured for 3–4 days in Dulbecco’s modified Eagle medium containing 10% FBS and 50 ng/ml murine thrombopoietin (R&D Systems) and purified using bovine serum albumin (BSA) gradients. After that, the MK-enriched (MK-CM) and non-MK (pre-MK-CM) fractions were collected and cultured in phenol-red-free α-MEM without FBS or thrombopoietin for 24 h. MK-CM and pre-MK-CM were collected by filtration through a 0.45-µm membrane filter and stored at −80 °C until use.

### Generation of *Vgf*^−/−^ mice

*Vgf*^−/−^ C57BL6 mice were generated and obtained from the Genetically Engineered Animal Research (GEAR) Core, Asan Medical Center (Seoul, Korea). As described previously, using Transcription activator-like effector nuclease (TALEN) methodology, *Vgf*-TALEN mRNA was injected into C57BL6 mouse embryos^18^. For genotyping, genomic DNA was extracted from tail tissues and subjected to PCR with a Solg Taq DNA Polymerase Kit (SolGent) with forward (5′-TCC TTC TAC TGA TCC AGG GGT-3′) and reverse (5′-GTC ATC CTT TGG CCG GGA C-3′) primers.

### Cell viability and proliferation assays

Murine preosteoblast MC3T3-E1 cells (5 × 10^3^ cells per well) were seeded into 96-well culture plates and incubated for 1–2 days. For the cell viability assay, 10 μl of Cell Counting Kit-8 solution (Dojindo) was added to each well, and the plates were incubated for 1 h. A microplate reader (Tecan Group) was used to measure the absorbance at 450 nm. For the cell proliferation assay, the cells were incubated with 5-bromo-2′-deoxyuridine (BrdU) for 24 h, and cell proliferation was analyzed using a BrdU labeling and detection kit (Roche) according to the manufacturer’s instructions.

### Bone nodule formation assay

After 14 days of treatment with 50 μg/ml ascorbic acid (Sigma-Aldrich) and 10 mM β-glycerophosphate (Sigma-Aldrich), MC3T3-E1 cells were allowed to differentiate into osteoblasts. The cells were fixed with 70% ethanol, stained with 40 mM Alizarin red S solution (Sigma-Aldrich) and washed with distilled water. After the bound Alizarin red S solution was dissolved in 10% cetylpyridinium chloride (Sigma-Aldrich), the eluted samples were quantified by measuring the absorbance at 570 nm using a microplate reader (Tecan Group).

### VGF-derived peptides and antibodies

Details are provided in the Supplementary Materials and Methods.

### Osteocalcin and AQEE30 ELISAs

MC3T3-E1 cells (2 × 10^5^ cells per well) were seeded in 12-well culture plates and differentiated into osteoblasts with 5 μM AQEE30 for 7 days. CM was collected and measured using an osteocalcin enzyme-linked immunosorbent assay (ELISA) kit (BT-470, Alfa Aesar). AQEE30 levels in pre-MK-CM and MK-CM were measured using an AQEE30 ELISA kit (Phoenix Pharmaceuticals) according to the manufacturer’s instructions.

### Cell cycle assay

MC3T3-E1 cells (1 × 10^5^ cells) were seeded into 35-mm culture dishes, cultured for 1 day, treated with 1 μM AQEE30 for 12 h before trypsinization, washed with PBS and fixed with 70% ethanol for 1 h. The cells were centrifuged at 1700 rpm for 5 min, washed with PBS and treated with 50 μg/ml RNase solution (Sigma-Aldrich) at room temperature for 15 min. The samples were then treated with 50 μg/ml propidium iodide solution (Sigma-Aldrich) at room temperature for 30 min and measured with a BD FACSCanto II flow cytometer (BD Biosciences).

### Microcomputed tomography

Femurs from *Vgf*^−/−^ and their wild-type (WT) littermates were fixed with 4% paraformaldehyde. The bone samples were scanned with a SkyScan 1172 system (SkyScan) at 70 kV, 126 μA and a 6.8-μm pixel size. The regions of interest extended 3 mm from the growth plate of each femur to the proximal metaphysis, and three-dimensional algorithms were used to determine the relevant morphometric parameters using CT-Analyser (version 1.16.1.0, CTAn, SkyScan) software.

### Immunofluorescence, dynamic histomorphometry and serum bone markers

For immunofluorescence staining, the dissected femur or calvaria bone tissues were fixed, decalcified and sectioned with a cryostat; the 5-μm-thick paraffin bone sections were deparaffinized, rehydrated and stained with anti-osteocalcin (1/200 dilution; Takara) and anti-periostin (1/100 dilution; Proteintech) antibodies for 16 h at 4 °C. The sections were subsequently washed and incubated with an Alexa Fluor 647 secondary antibody (1/400 dilution; Invitrogen) for 1 h at room temperature. Nuclei were counterstained with DAPI, and fluorescence was detected using a confocal microscope (LSM710, Carl Zeiss). Image analysis was performed on the area beneath the growth plate of the distal metaphysis of the femur. For dynamic histomorphometry, *Vgf*^−/−^ mice and their WT littermates were injected with calcein (30 mg/kg; Sigma-Aldrich) 5 and 3 days before they were euthanized. The femurs were cut into 20-μm-thick sections, and the bone sections were photographed and analyzed with a semiautomatic image analysis system (Histometry RT Digitizer, System Supply) and the Bio-Quant program (version 19.9.60, Bio-Quant). For the serum bone marker analysis, the serum osteocalcin concentration was measured using an osteocalcin ELISA kit (BT-470, Alfa Aesar).

### In vivo calvarial bone formation

The calvarial bone formation assay was performed according to a previous report with minor modifications^13^. Five-week-old male C57BL/6N mice were injected subcutaneously over the right parietal calvarial bone with AQEE30 or PEG_2_-AQEE30 at a dose of 0.5 mg/kg in 50 μl of PBS daily for 5 days. After 16 days, all the mice were euthanized, and the calvaria were collected. Calvaria were fixed with 4% paraformaldehyde for 24 h and decalcified in Osteosoft solution (Sigma-Aldrich, 1.01728.1000) for 2 weeks. Paraffin-embedded sections (5 μm thick) of calvaria were stained with hematoxylin and eosin (H&E) or processed for immunostaining of osteocalcin as described above. Images were acquired with an Olympus VS200 slide scanner (Olympus) and analyzed with Olympus OlyVIA software (version 3.3, Olympus). Calvaria bone width was evaluated as lengths that included both old and new bones. The calvaria bone width was determined by averaging four lengths measured at equal intervals from 900 to 1700 μm from the sagittal suture. The total bone area was determined as the area between the sagittal suture and the muscle attachment site. The new bone area was measured on the periosteal surface of the calvaria in the same region as the total bone area. Because new bone is distinguished by its lighter color compared with old bone, the areas of new and old bone were measured separately^19^. Width and bone area measurements were analyzed using the Image-Pro Plus program (version 6.0, Media Cybernetics).

### Mouse femoral defect model

Female C57BL/6 mice at 8 weeks of age were purchased from Orient Bio and equally divided into three groups: the control, AQEE30 and PEG_2_-AQEE30 groups. After anesthesia, the hair on the thigh of each mouse was removed and disinfected. The anterior skin of the femur was excised, and then the muscles were cut to expose the surface of the femur. A 0.8-mm hole was made in the femur using a drill. Saline solution was used to remove residual bone and avoid thermal necrosis. A collagen membrane (diameter of 1.5 mm; Geistlich Bio-Gide, Geistlich Pharma AG) was loaded with 100 μl of PBS (control), 3 μg of AQEE30 and 3 μg of PEG_2_-AQEE30 and transplanted into the femoral defect site. Finally, the incised skin and muscle were closed with 4–0 nylon sutures. After 10 days, the femurs were collected and fixed in 4% paraformaldehyde for 24 h, after which microcomputed tomography (micro-CT) was performed. The area of the bone defect was quantified using the ImageJ program (version 1.54f, National Institutes of Health).

### RNA interference, real-time PCR and western blotting

*VGF* and *C3AR1* short interfering RNAs (siRNAs) (listed in Supplementary Table 1) were purchased from Qiagen and Bioneer, respectively. MC3T3-E1 cells were transfected with 20 pmol of siRNAs for 24 h using Lipofectamine 2000 (Invitrogen) according to the manufacturer’s instructions. Total RNA was isolated using TRIzol (Invitrogen), and cDNA was synthesized using the SuperScript III First-Strand Synthesis System (Invitrogen). Quantitative real-time PCR (qRT‒PCR) was performed using SYBR Green I Master Mix (Roche) according to the manufacturer’s instructions, and the primers used for qRT‒PCR are listed in Supplementary Table 2. TaqMan probes (Mm00443947_m1, Mm00726334_s1 and Mm03928990_g1, Invitrogen) were used to determine the expression levels of *CDK2*, *CDK4* and *18S*, respectively.

For western blotting, the cells were lysed with RIPA buffer (50 mM Tris–HCl pH 7.4, 150 mM NaCl, 1 mM EDTA, 1% NP40, 1% sodium dodecyl sulfate (SDS), 1 mM phenylmethylsulfonyl fluoride, 1 mM Na_3_VO_4_ and 1 mM glycerol phosphate) that contained a protease inhibitor cocktail (Sigma-Aldrich). The cell lysates were centrifuged at 13,000 rpm for 10 min, and the supernatants were denatured with SDS sample buffer (62.5 mM Tris–HCl pH 6.8, 2.5% SDS, 10% glycerol, 0.002% bromophenol blue and 5% mercaptoethanol) and subjected to SDS polyacrylamide gel electrophoresis. The separated proteins were transferred onto nitrocellulose membranes (Amersham Pharmacia Biotech) for probing with primary and horseradish peroxidase (HRP)-conjugated secondary antibodies. All the blots were developed with an enhanced chemiluminescence kit (PerkinElmer), and the intensities of the western blotting bands were quantified using ImageJ (version 1.52a, National Institutes of Health).

### AQEE30 and C3AR1-binding ELISA

The binding ELISA was performed as described previously^20^. MC3T3-E1 cell lysates were added to 10 μg/ml AQEE30- or BSA-coated binding ELISA 96-well plates (Thermo Scientific) for 2 h at room temperature. The plates were washed, blocked with 1% BSA and then incubated with the C3AR1 antibody (Biorbyt) for 2 h. After washing, an HRP-conjugated secondary antibody (Cell Signaling Technology) was added for 2 h. The HRP–3,3′,5,5′-tetramethylbenzidine system generated the colorimetric signal. The absorbance was read at 450 nm using an ELISA microplate reader (Tecan Group).

### Peptide pulldown assay

The peptide pulldown assay was performed as described previously^21^. His-tagged C3AR1 (Sino Biological, HG11469-CH) was expressed in HEK293T cells (American Type Culture Collection), and cell lysates were prepared with RIPA buffer, mixed with 10 μg and 30 μg of biotinylated AQEE30 peptide that had previously bound to streptavidin agarose beads (Thermo Scientific) at 4 °C in binding buffer (20 mM Tris–HCl pH 7.3, 150 mM KCl, 0.2 mM EDTA, 20% glycerol and 0.1% Nonidet P-40) and incubated overnight. The beads were washed, and the bound proteins were analyzed by western blotting with an anti-His antibody.

### cAMP ELISA and protein kinase A activity assay

MC3T3-E1 cells were pretreated with SQ22536 (Sigma-Aldrich, S153), H89 (Sigma-Aldrich, B1427) or SB290157 (Sigma-Aldrich, SML1192) for 1 h, and 1 or 5 μM AQEE30 was added for 30 min. Cell lysates were lysed with 100 μl of lysis buffer (Enzo Laboratories). After centrifugation at 13,000 rpm for 10 min, the supernatant was analyzed with a cAMP ELISA kit (Enzo Life Sciences) according to the manufacturer’s instructions. The absorbance was measured at 405 nm using a microplate reader (Tecan Group). For the protein kinase A (PKA) activity assay, total cell lysates were prepared and examined using a PKA activity assay kit (Enzo Life Sciences) according to the manufacturer’s instructions, and the absorbance at 450 nm was measured using a microplate reader (Tecan Group).

### Quantification and statistical analysis

All quantitative data are presented as the mean ± s.e.m. Intergroup differences were examined using Student’s two-tailed *t*-test, and multigroup differences were ascertained using one-way analysis of variance followed by the Bonferroni multiple-comparisons test. All the statistical analyses were performed in GraphPad Prism (version 8.0, GraphPad Software), and *P* < 0.05 was considered statistically significant.

## Results

### VGF and VGF-derived AQEE30 peptide from MK-CM stimulate bone formation in vitro

**Fig. 1:**
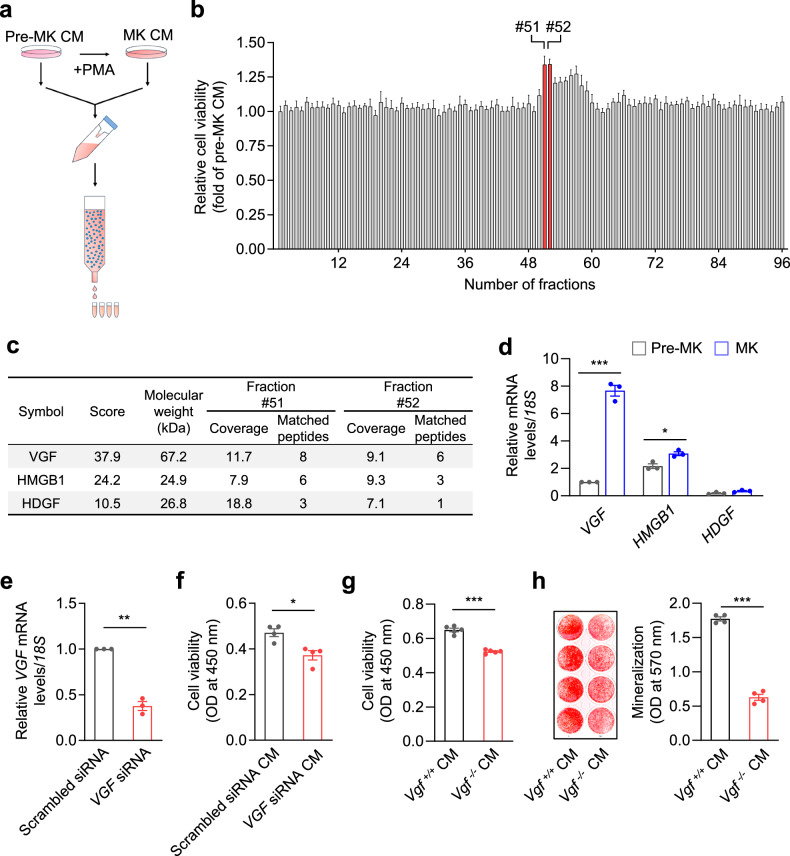
VGF from MK-CM induces osteoblast proliferation and bone formation in vitro. **a**, Secretion fractionation protocol. Premature K562 cells (pre-MKs) were treated with PMA and then differentiated into mature MK-like cells for 3 days. CM of pre-MKs and MKs was collected, concentrated and then divided into 96 fractions by size-exclusion chromatography. **b**, The viability of MC3T3-E1 cells after treatment with each pair of pre-MK-CM and MK-CM was measured using a Cell Counting Kit-8 (*n* = 2). **c**, Secreted proteins commonly identified in fractions 51 and 52 of the MK-CM. **d**, Real-time PCR analysis of *VGF*, *HMGB1* and *HDGF* in pre-MKs and MKs (*n* = 3). **e**, K562 cells were transfected with scrambled siRNA or *VGF* siRNA, and the knockdown efficiency was measured by real-time PCR (*n* = 3). **f**, K562 cells were transfected with scrambled siRNA and *VGF* siRNA and then differentiated into mature MKs. MC3T3-E1 cells were treated with their CM, and cell viability was measured (*n* = 4). **g**, Murine fetal liver cells isolated from *Vgf*^−/−^ mice and their WT littermates were differentiated into mature MKs by thrombopoietin treatment. The viability of MC3T3-E1 cells after treatment with CM from mature MKs was measured (*n* = 5). **h**, CM was collected as described in **g**. Bone nodule formation in MC3T3-E1 cells was tested after treatment with CM from mature MKs. Mineralized samples were stained with Alizarin red S (right) and measured at 570 nm (left, *n* = 4). The data represent the mean ± s.e.m. *P < 0.05, **P < 0.01, ***P < 0.001 versus the control groups.

To identify a novel bone-forming factor from MK-CM, we used a fractionated secretome strategy. The CM from differentiated MK-like and undifferentiated (K562) cells was separated into 96 paired fractions (Fig. 1a). Each fraction’s ability to stimulate osteoblast viability was tested (Fig. 1b), and two paired fractions with the greatest difference in this stimulant ability were selected. Using liquid chromatography–tandem mass spectrometry, 12 proteins were discovered (Fig. 1c and Supplementary Tables 3 and 4). As 3 of the 12 proteins can be secreted extracellularly, we performed qRT‒PCR to examine the mRNA expression levels of three genes, *VGF*, *HMGB1* and *HDGF*. Compared with their expression levels in undifferentiated cells, *VGF* was more differentially expressed and strongly expressed in MK-like cells, which indicates that VGF may be a significant factor that modulates cell viability (Fig. 1d).

To test the effect of *VGF* depletion in mature MK-like cells, we evaluated the effect of VGF siRNA on osteoblast viability (Fig. 1e). When MC3T3-E1 cells were treated with *VGF*-depleted MK-CM, osteoblast viability decreased significantly (Fig. 1f), which was confirmed in *Vgf*-knockout mice generated using TALEN technology (Supplementary Fig. 1a,b). Compared with CM from their WT littermates, CM from MK-like cells from *Vgf*^−/−^ mice significantly decreased the viability and mineralization of MC3T3-E1 cells (Fig. 1g,h). The CM also decreased the viability and mineralization of primary periosteal skeletal stem cells (PSSCs) (Supplementary Fig. 2a,b) but had no effect on colony formation or chondrogenic differentiation (Supplementary Fig. 2c,d). Thus, VGF secreted from MK-CM may crucially regulate osteoblast growth and mineralization in vitro.

**Fig. 2:**
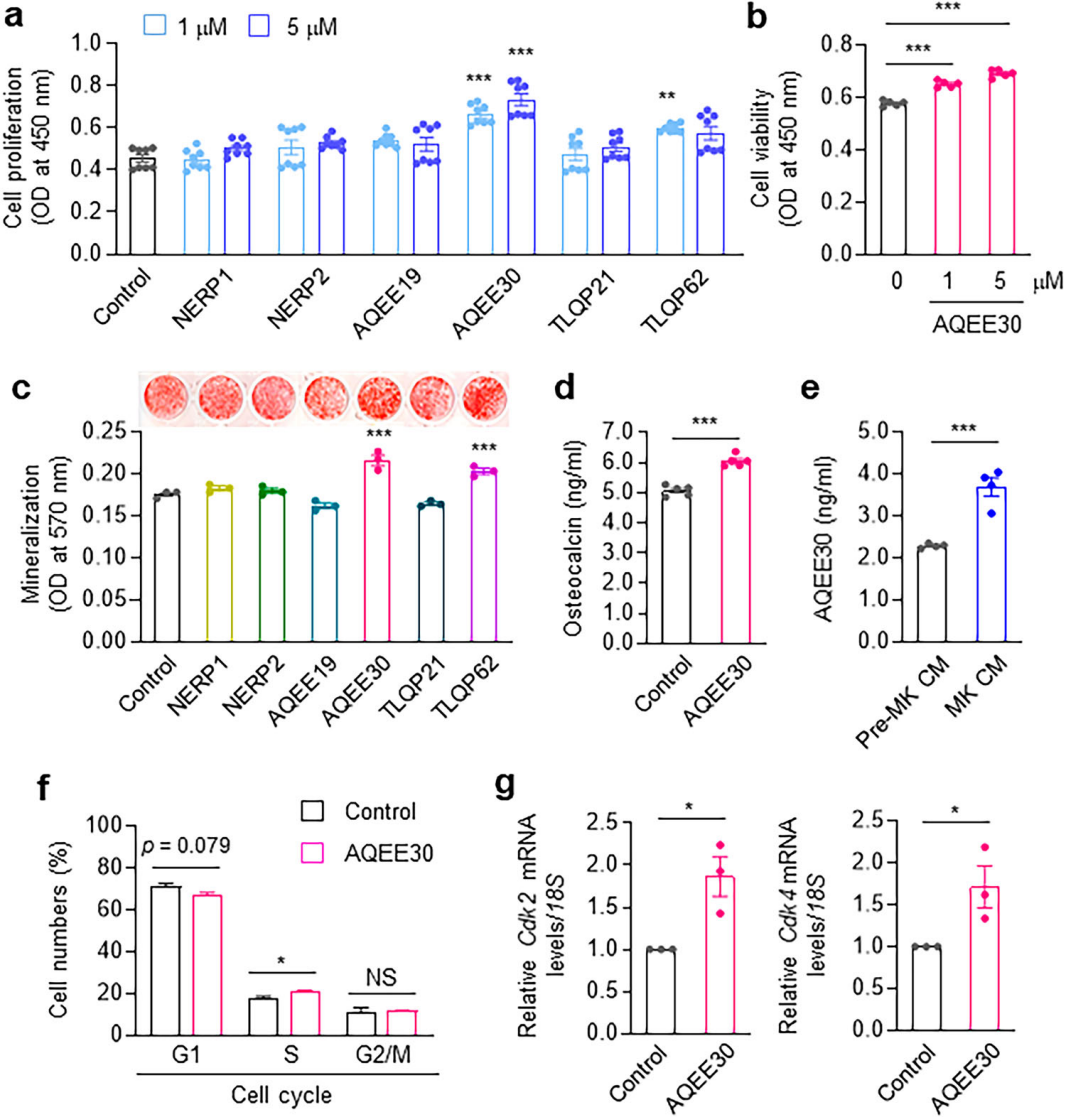
The VGF-derived peptide AQEE30 promotes osteoblast proliferation and bone mineralization. **a**, MC3T3-E1 cells were treated with several VGF-derived peptides for 48 h, and a BrdU assay was performed (*n* = 8). **b**, MC3T3-E1 cells were treated with AQEE30 for 48 h, and cell viability was determined (*n* = 5). **c**, A bone nodule formation assay was carried out as described in **a** using 5 μM VGF-derived peptides. Bone mineralization was visualized with Alizarin red S (top) and measured at 570 nm (bottom, *n* = 3). **d**, After treatment with 5 μM AQEE30 for 7 days in osteogenic culture medium, osteocalcin levels in MC3T3-E1 cells were measured by ELISA (*n* = 5). **e**, CM was collected from pre-MKs and MKs, and an AQEE30 ELISA was performed (*n* = 4). **f**, Cell cycle distribution was measured using propidium iodide staining after MC3T3-E1 cells were treated with 1 μM AQEE30 (*n* = 3). **g**, Real-time PCR analysis of *Cdk2* and *Cdk4* in MC3T3-E1 cells treated with or without 1 μM AQEE30 (*n* = 3). The data represent the mean ± s.e.m. *P < 0.05, ***P < 0.001 versus untreated or pre-MK-CM-treated control. NS, not significant.

As the VGF protein is proteolytically cleaved into several biologically active peptides^22^, we evaluated the effects of various VGF-derived peptides on osteoblast proliferation and mineralization activities. The AQEE30 peptide significantly increased osteoblast proliferation, whereas the other peptides did not (Fig. 2a). AQEE30 consistently and dose-dependently promoted the viability of MC3T3-E1 cells (Fig. 2b) and PSSCs (Supplementary Fig. 2e) and stimulated their mineralization (Fig. 2c and Supplementary Fig. 2f) and/or osteocalcin secretion (Fig. 2d). The AQEE30 peptide had no effect on colony formation or chondrogenic differentiation in PSSCs (Supplementary Fig. 2g,h). ELISAs comparing AQEE30 levels in CM from differentiated MK-like and undifferentiated cells revealed higher AQEE30 levels in CM from MK-like cells (Fig. 2e).

To further elucidate the effects of AQEE30 on osteoblast proliferation, we performed cell cycle analysis. AQEE30 tended to decrease the number of cells in the G0–G1 phase and significantly increased the number of cells in the S phase (Fig. 2f). Cyclin-dependent kinases (CDKs) are required for the G1/S phase transition to mitosis^23^. *Cdk2* and *Cdk4* mRNA levels increased significantly with AQEE30 treatment (Fig. 2g), suggesting that AQEE30 may increase *Cdk2* and *Cdk4* expression in osteoblasts, resulting in cell cycle progression and increased proliferation. These results indicate that MK increases the expression of *Vgf* mRNA and AQEE30 according to their degree of differentiation and thereby induces paracrine stimulation of osteoblastic proliferation and bone formation.

### VGF loss induces an osteopenic phenotype in cortical bone by suppressing bone formation

**Fig. 3:**
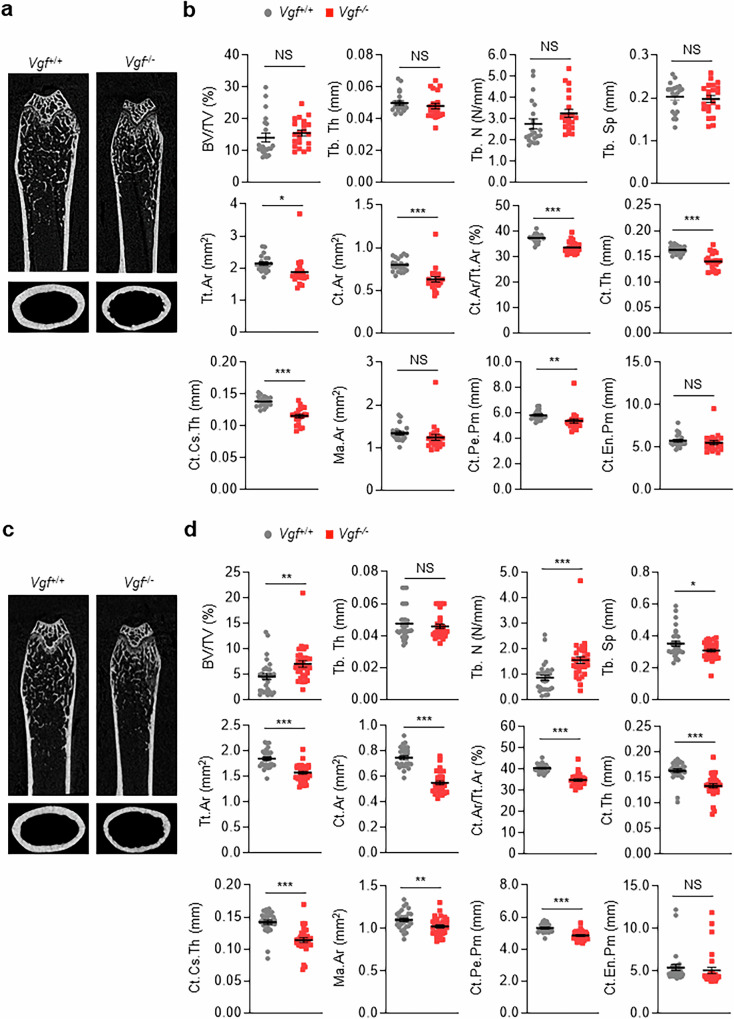
Osteopenic phenotypes of cortical bone in *Vgf*^−/−^ mice. **a**–**d**, Micro-CT of the proximal femurs of 16-week-old male (**a** and **b**) and female (**c** and **d**) *Vgf*^−/−^ mice and their WT littermates. Micro-CT images (**a** and **c**) and quantitative assessments of their trabecular and cortical bone parameters (**b** and **d**) are shown. The BV/TV, Tb.Th, Tb.N, Tb.Sp, Tt.Ar, Ct.Ar, Ct.Th, Ct.Cs.Th, marrow area (Ma.Ar), Ct.Pe.Pm and cortical endocortical perimeter (Ct.En.Pm) are shown. The data are presented as the mean ± s.e.m. (21 *Vgf*^+/+^ and 21 *Vgf*^−/−^ male mice and 29 *Vgf*^+/+^ and 31 *Vgf*^−/−^ female mice). *P < 0.05, **P < 0.01, ***P < 0.001 versus *Vgf*^+/+^ mice. NS, not significant.

Compared with their WT littermates, both male and female *Vgf*^−/−^ mice had significantly lower body weights and shorter femur lengths at 8 and 16 weeks (Supplementary Fig. 3a–c). Micro-CT analyses of male mice revealed similar trabecular bone parameters, such as the trabecular bone volume (BV/TV), trabecular number (Tb.N), trabecular thickness (Tb.Th) and trabecular separation (Tb.Sp), in male *Vgf*^−/−^ mice and their WT littermates (Fig. 3a,b). However, male *Vgf*^−/−^ mice presented significantly lower cortical bone mass parameters, including cortical thickness (Ct.Th), cortical thickness per total area (Ct.Ar/Tt.Ar) and cortical cross-sectional thickness (Ct.Cs.Th), than their WT littermates. Moreover, the cortical periosteal perimeter (Ct.Pe.Pm) decreased in the *Vgf*^−/−^ mice.

Female *Vgf*^−/−^ mice presented similar cortical bone alterations (that is, lower Ct.Th, Ct.Ar/Tt.Ar, Ct.Cs.Th and Ct.Pe.Pm; Fig. 3c,d). By contrast, the trabecular bone mass increased in female *Vgf*^−/−^ mice: the BV/TV and Tb.N significantly increased, whereas the Tb.Sp decreased. These findings indicate that *Vgf* loss sex-nonspecifically decreases cortical bone mass but sex-specifically affects trabecular bone.

**Fig. 4:**
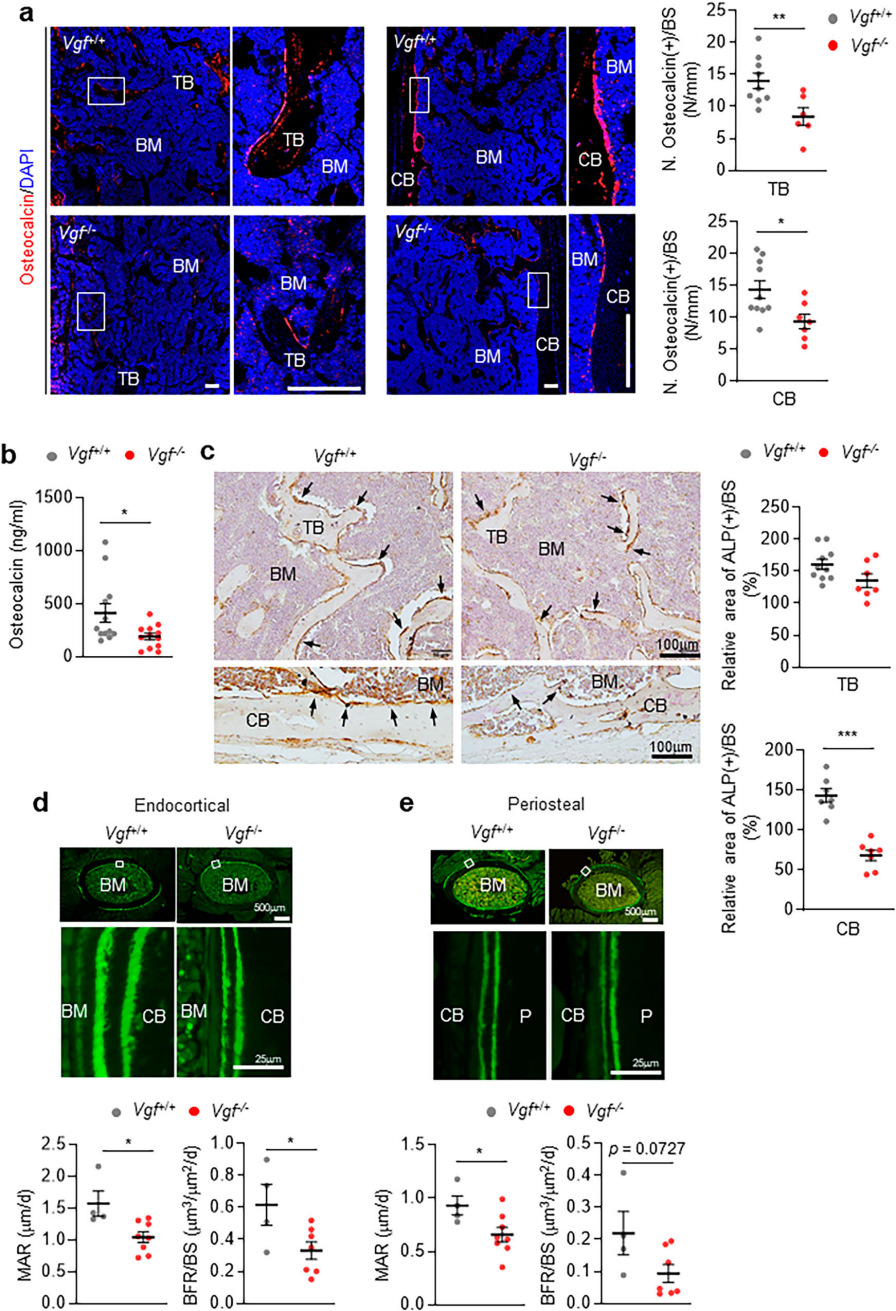
Decreased cortical bone formation in *Vgf*^−/−^ mice. **a**, Numbers of osteocalcin-positive cells in 16-week-old male *Vgf*^−/−^ mice (*n* = 6 for TB and *n* = 7 for CB) and their WT littermates (*n* = 9 for TB and *n* = 10 for CB). Representative images are shown on the left, and their quantitative results are shown on the right. The right panels show enlarged views of the white boxes. Scale bar, 100 μm. **b**, Osteocalcin levels in the sera of 16-week-old male *Vgf*^−/−^ mice (*n* = 12) and their WT littermates (*n* = 12). **c**, Representative ALP immunohistochemistry images of the femurs of 16-week-old male *Vgf*^*−/−*^ mice (*n* = 7 for TB and CB) and their WT littermates (*n* = 10 for TB and *n* = 7 for CB) are shown on the left. The relative areas of ALP per TB and CB surfaces are shown on the right. The arrows indicate ALP-positive areas. **d**,**e**, Dynamic histomorphometry of 8-week-old female *Vgf*^−/−^ mice (*n* = 7) and their WT littermates (*n* = 4). **d**,**e**, Mineral apposition rate (MAR) and bone formation rate per bone surface (BFR/BS) on the endocortical (**d**) and periosteal (**e**) bone surfaces. Representative calcein staining images are shown in the top (low-magnification images) and middle (high-magnification images) panels. The middle panels show enlarged views of the white boxes in the top panels. The quantification results are shown in the bottom panels. P, periosteum; BM, bone marrow; BS, bone surface; CB, cortical bone; TB, trabecular bone. The data represent the mean ± s.e.m. *P < 0.05, **P < 0.01 versus *Vgf*^+/+^ mice.

We then counted the number of osteoblasts. The numbers of osteocalcin-positive cells decreased significantly in both the trabecular and cortical bones of *Vgf*^−/−^ mice (Fig. 4a). The serum osteocalcin level decreased in the *Vgf*^−/−^ mice (Fig. 4b). The alkaline phosphatase (ALP)-positive area also decreased in the cortical bones of *Vgf*^−/−^ mice (Fig. 4c). However, the difference in the ALP-positive area was not statistically significant between the trabecular bones of *Vgf*^−/−^ mice and their WT littermates (Fig. 4c), suggesting developmental stage-dependent differences in the number of bone-forming cells. Double-calcein labeling analysis of bone revealed a decreased mineral apposition rate in both the endocortical (Fig. 4d) and periosteal (Fig. 4e) areas of *Vgf*^−/−^ mice compared with their WT littermates. The bone formation rate significantly or marginally decreased in both areas. These findings suggest that *Vgf* loss may decrease cortical bone mass, at least partially, by suppressing bone formation, particularly in cortical bones. Unlike in cortical bone, there were minimal changes in the trabecular bones of *Vgf*^−/−^ mice (Fig. 3). We examined periostin^24^ and osteocytes^25^, which are particularly involved in cortical bone mass. The expression of periosteal periostin (Supplementary Fig. 4a) and the number of cortical osteocytes (Supplementary Fig. 4b) were lower in *Vgf*^−/−^ mice than in their WT littermates.

### AQEE30 stimulates osteoblast proliferation and bone formation via cAMP upregulation

**Fig. 5:**
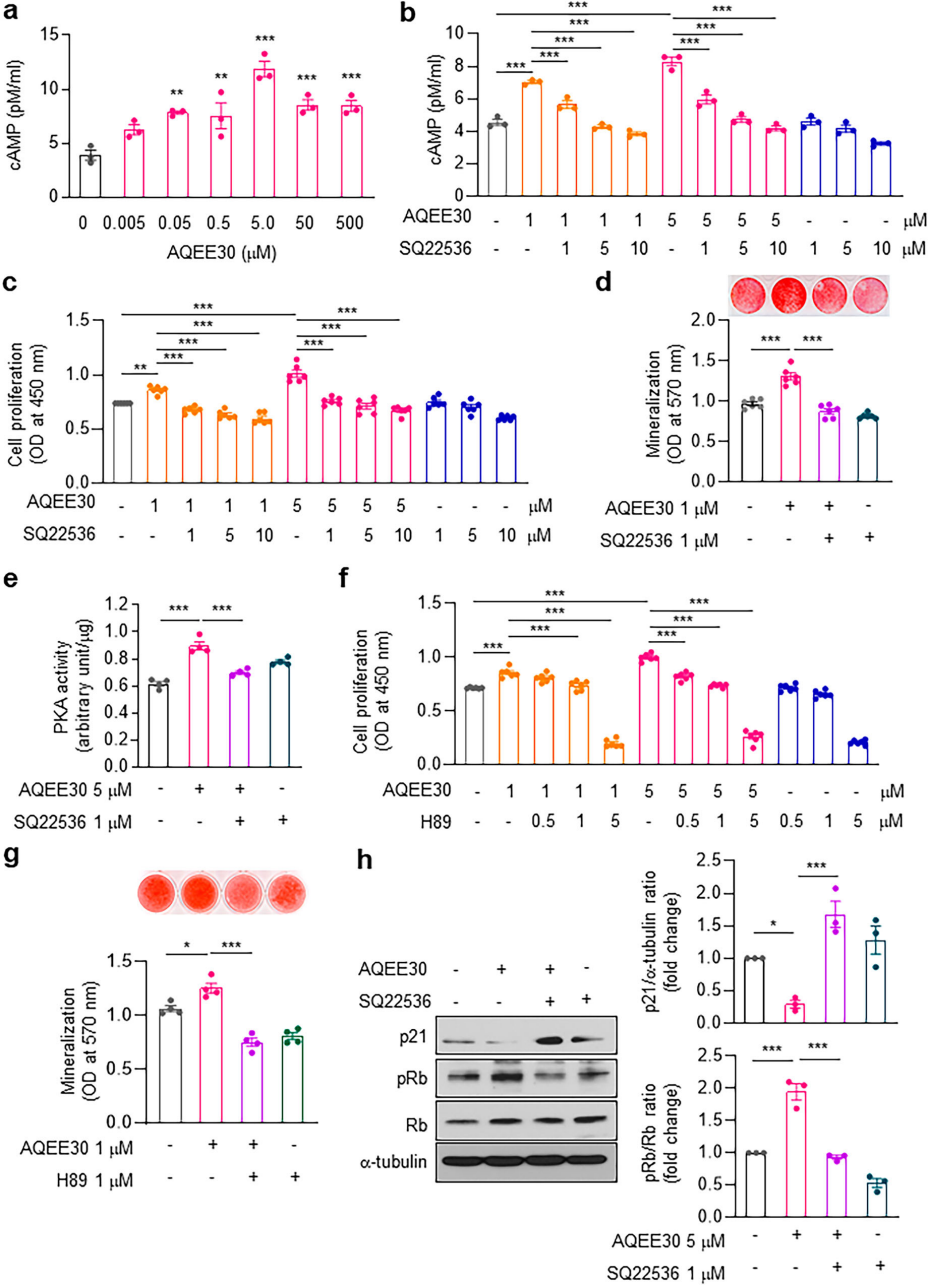
AQEE30 regulates osteoblast proliferation and bone formation through the cAMP‒PKA signaling pathway. **a**, MC3T3-E1 cells were treated with the indicated concentrations of AQEE30 and subjected to a cAMP ELISA (*n* = 3). **b**, MC3T3-E1 cells were treated with 1 or 5 μM AQEE30 in the presence or absence of the indicated doses of SQ22536. Whole-cell lysates were analyzed in a cAMP assay (*n* = 3). **c**, After MC3T3-E1 cells were treated as described in **b**, cell proliferation was analyzed in a BrdU assay (*n* = 6). **d**, MC3T3-E1 cells were generated as described in **b**, and a bone nodule formation assay was performed. Mineralization was determined with Alizarin red S (upper) staining, and the absorbance was measured at 570 nm (bottom, *n* = 6). **e**, MC3T3-E1 cells were treated with AQEE30 in the presence or absence of SQ22536, and PKA activity was analyzed (*n* = 4). **f**,**g**, MC3T3-E1 cells were treated with 1 or 5 μM AQEE30 in the presence or absence of the indicated doses of H89 and then analyzed in a BrdU assay (**f**, *n* = 6), and bone nodule formation was determined (**g**, *n* = 4). **h**, After MC3T3-E1 cells were treated with AQEE30 in the presence or absence of SQ22536, western blotting for p21, phospho-Rb (pRb) and Rb was performed. The relative band densities normalized to those of α-tubulin and Rb are shown (*n* = 3). The data represent the mean ± s.e.m. *P < 0.05, **P < 0.01, ***P < 0.001 versus untreated control or between the groups.

We next investigated the downstream targets of the VGF-derived peptide AQEE30 in osteoblasts. Runx2 and β-catenin are essential signaling molecules for osteoblastic bone formation^26,27^, but we failed to demonstrate any significant effects of AQEE30 on the transcriptional activities of Runx2 and β-catenin (Supplementary Fig. 5a,b). As cAMP–PKA signaling also stimulates bone formation^28^, we measured intracellular cAMP levels. AQEE30 dose-dependently increased cAMP levels in MC3T3-E1 cells, with a maximal effect at 5 μM (Fig. 5a). The restoration of cAMP levels by the adenylyl cyclase inhibitor SQ22536 (Fig. 5b) almost completely blocked AQEE30-stimulated osteoblastic proliferation (Fig. 5c) and mineralization (Fig. 5d).

Next, the activity of PKA, a downstream signal of cAMP, was measured in MC3T3-E1 cells. AQEE30 increased PKA activity, which was reversed with SQ22536 pretreatment (Fig. 5e). Pretreatment with the PKA inhibitor H89 blocked AQEE30-stimulated osteoblastic proliferation (Fig. 5f) and mineralization (Fig. 5g). These findings indicate that cAMP–PKA signaling mediates the effects of AQEE30 on osteoblastic proliferation and bone formation.

The CDK inhibitor p21, a marker of cell cycle arrest^29^, dephosphorylates retinoblastoma (Rb), which regulates cell cycle progression^30^. The results from the western blot analysis revealed that AQEE30 decreased p21 expression and increased Rb phosphorylation (Fig. 5h), whereas SQ22536 pretreatment almost completely blocked both the AQEE30-induced changes in expression, suggesting that cAMP–PKA signaling may mediate cell cycle progression by AQEE30 via Rb phosphorylation.

### C3AR1 as a putative receptor for AQEE30

**Fig. 6:**
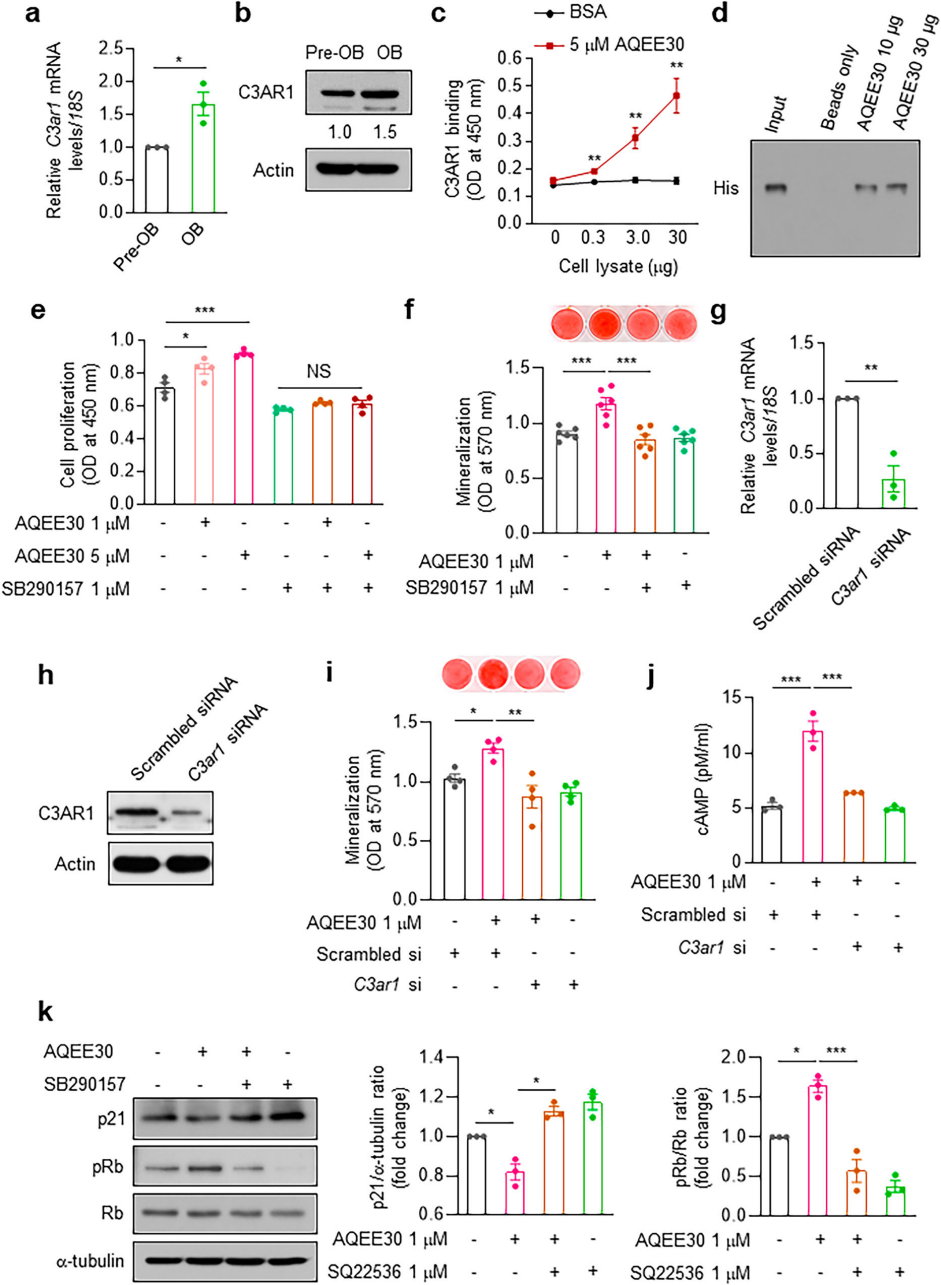
C3AR1 is a putative receptor for AQEE30 in osteoblasts. **a**, The expression of *C3ar1* mRNA in MC3T3-E1 cells before (pre-OB) and after (OB) differentiation into osteoblasts for 7 days in osteogenic culture medium. The mRNA levels were analyzed by real-time PCR (*n* = 3). **b**, MC3T3-E1 cells were generated as described in **a**, and total cell lysates were analyzed by western blotting with an anti-C3AR1 antibody. **c**, MC3T3-E1 cell lysates at the indicated doses were seeded onto ELISA plates coated with 5 μM AQEE30 for 2 h. The wells were further incubated with an anti-C3AR1 antibody and quantitatively analyzed by incubation with an HRP-conjugated secondary antibody. The absorbance was measured at 450 nm on a microplate reader (*n* = 4). **d**, The indicated biotin-labeled AQEE30 peptides were incubated with His-tagged C3AR1 expressed in HEK293T cells and then immunoprecipitated with streptavidin agarose beads. The precipitates were analyzed by immunoblotting with an anti-His antibody. The input represents 5% of the total cell lysates used for immunoprecipitation. **e**, MC3T3-E1 cells were treated with the indicated doses of AQEE30 in the presence or absence of SB290157, and cell viability was measured (*n* = 4). **f**, MC3T3-E1 cells were treated with AQEE30 in the presence or absence of SB290157 and analyzed in a bone nodule formation assay. Mineralization was determined with Alizarin red S staining (top), and the absorbance was measured at 570 nm (bottom, *n* = 6). **g**,**h**, MC3T3-E1 cells were transfected with scrambled siRNA and *C3ar1* siRNA. The knockdown efficiency of *C3ar1* was analyzed by real-time PCR (**g**, *n* = 3) and western blotting (**h**). Actin was used as a loading control. **i**, MC3T3-E1 cells were transfected with scrambled and *C3ar1* siRNAs in the presence or absence of AQEE30 and subjected to a bone nodule formation assay (*n* = 4). **j**, After MC3T3-E1 cells were treated as described in **i**, a cAMP assay was performed (*n* = 3). **k**, MC3T3-E1 cells were generated as described in **e**, and western blotting was carried out with the indicated antibodies against p21, phospho-Rb (pRb) and Rb; their relative band densities, normalized to those of α-tubulin and Rb, respectively, are shown on the right (*n* = 3). The data represent the mean ± s.e.m. *P < 0.05, **P < 0.01, ***P < 0.001 versus the control groups or between the groups. NS, not significant.

G-protein-coupled receptors activate adenylyl cyclases, leading to cAMP stimulation^31^. C3AR1, a G-protein-coupled receptor, is one of the receptors for TLQP21, another VGF-derived peptide^32,33^; furthermore, stimulated C3AR1 increases the level of intracellular cAMP^34^. First, we measured C3AR1 expression in osteoblasts and found higher mRNA (Fig. 6a) and protein (Fig. 6b) expression levels in differentiated osteoblasts than in immature preosteoblasts. Binding ELISA revealed a dose-dependent interaction between AQEE30 and C3AR1 in MC3T3-E1 cells (Fig. 6c), which was confirmed by immunoprecipitation (Fig. 6d).

C3AR1 activity and expression were suppressed by a chemical inhibitor and its siRNA, respectively. The viability (Fig. 6e) and mineralization (Fig. 6f) of the AQEE30-stimulated osteoblasts were completely restored by the C3AR1 inhibitor SB290157. Pretreatment with *C3ar1* siRNA (Fig. 6g,h) blocked AQEE30-stimulated mineralization (Fig. 6i) and intracellular cAMP (Fig. 6j). The AQEE30-mediated suppression of p21 expression and AQEE30-stimulated Rb phosphorylation were reversed by pretreatment with a C3AR1 inhibitor (Fig. 6k). These findings suggest that C3AR1 may be a receptor for AQEE30 in osteoblasts.

### PEGylated AQEE30 promotes osteoblast proliferation and bone formation in vitro and in vivo

**Fig. 7:**
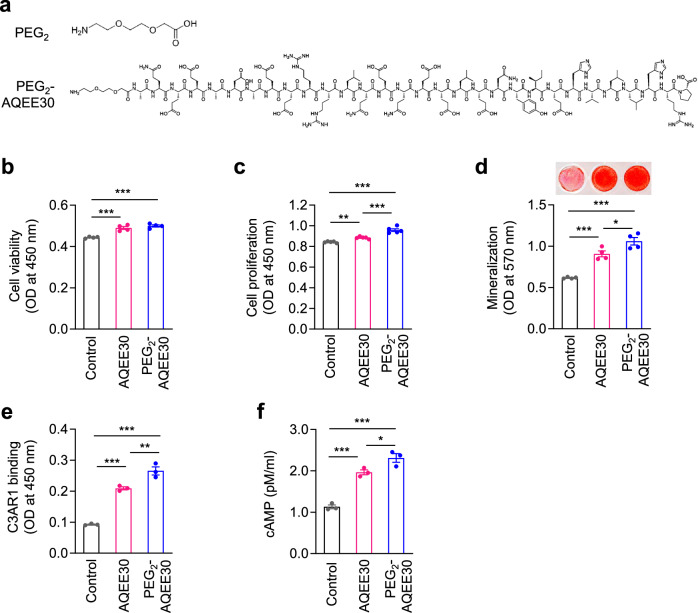
Effects of mini-PEGylated AQEE30 on bone formation in vitro. **a**, The structures of mini-PEG_2_ (PEG_2_) and mini-PEGylated AQEE30 (PEG_2_-AQEE30). **b**–**d**, MC3T3-E1 cells were treated with 5 μM AQEE30 or PEG_2_-AQEE30 for 48 h, after which cell viability (**b**, *n* = 4), BrdU (**c**, *n* = 5) and mineralization (**d**, *n* = 4) assays were performed. Mineralized samples were stained with Alizarin red S (upper) and measured at 570 nm. **e**, MC3T3-E1 lysates (30 μg) were seeded onto ELISA plates coated with 5 μM AQEE30 or PEG_2_-AQEE30 for 2 h. The wells were further incubated with an anti-C3AR1 antibody, and the absorbance was measured at 450 nm using a microplate reader (*n* = 3). **f**, MC3T3-E1 cells were treated with 1 μM AQEE30 or PEG_2_-AQEE30 for 30 min. Whole-cell lysates were analyzed in a cAMP assay (*n* = 3). The data represent the mean ± s.e.m. *P < 0.05, **P < 0.01, ***P < 0.001 versus the control.

To increase stability^35,36^, we generated a mini-PEGylated AQEE30, PEG_2_-AQEE30 (Fig. 7a). The PEG_2_-AQEE30 peptide enhanced the viability of osteoblasts to a similar extent as did AQEE30 (Fig. 7b) and enhanced their proliferation (Fig. 7c) and mineralization activity (Fig. 7d) more potently than did AQEE30. Furthermore, binding ELISA also revealed that PEG_2_-AQEE30 had stronger binding activity to the C3AR1 receptor than did AQEE30 (Fig. 7e). Consistently, PEG_2_-AQEE30 stimulated greater cAMP secretion than did AQEE30 (Fig. 7f).

**Fig. 8:**
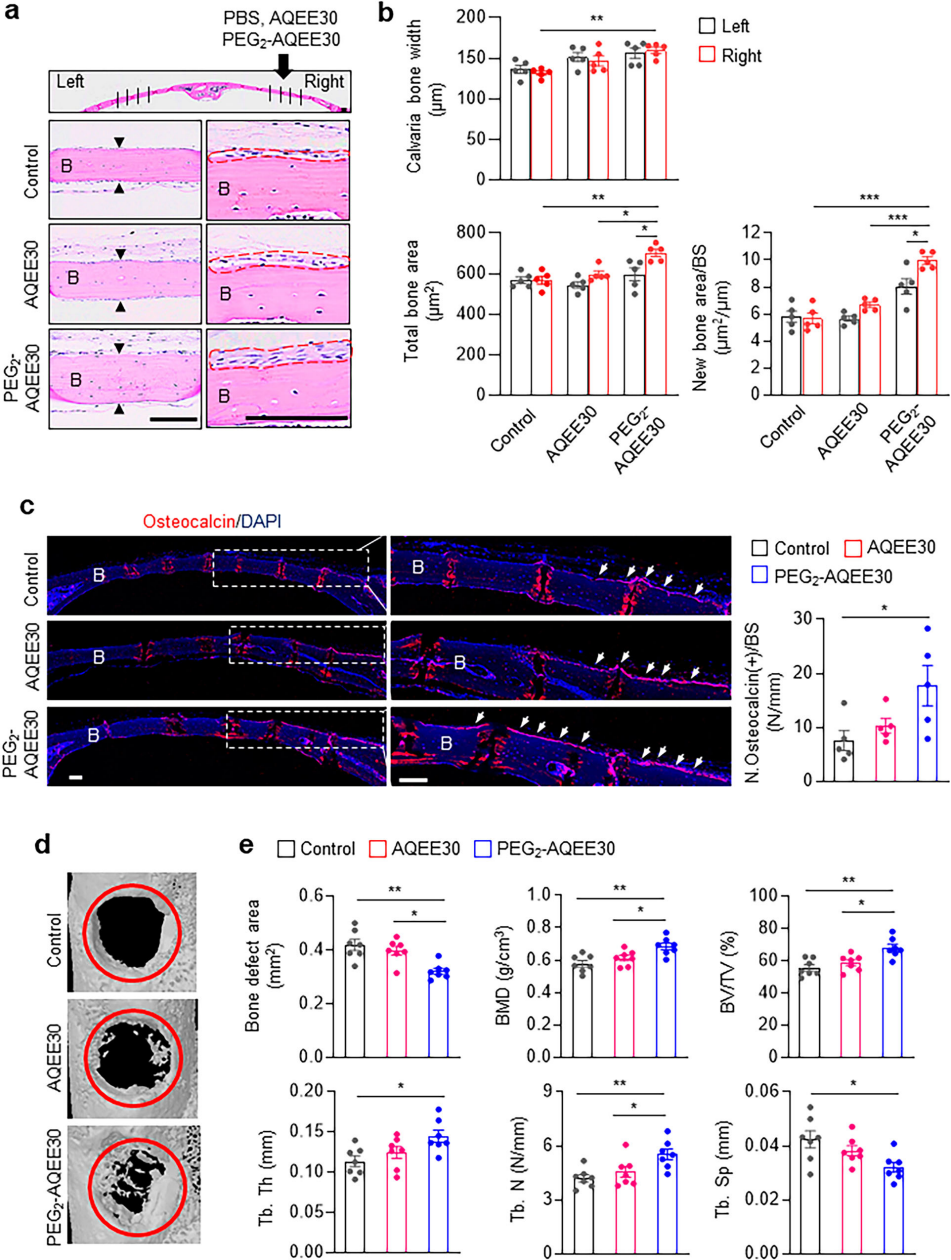
Therapeutic efficacy of mini-PEGylated AQEE30 in vivo. **a**, Five-week-old male C57BL/6N mice were injected with AQEE30 or PEG_2_-AQEE30 on the right side of the calvaria as described in the ‘Materials and methods’ section. PBS was injected as a negative control. H&E staining was performed on calvarial sections. The black arrowheads and red dotted lines indicate the calvaria bone surface and new bone area, respectively. Scale bar, 100 μm. B, calvaria bone. **b**, Calvaria bone width was quantified as the average of the four vertical lines shown in **a**. The total and new bone areas per bone surface (BS) were calculated from the left H&E-stained images (*n* = 5). **c**, Calvarial bone sections were subjected to osteocalcin immunostaining. Representative images of osteocalcin immunostaining (red) are shown in the left and middle panels. The middle panels show enlarged views of the white boxes. The number of osteocalcin-positive cells (white arrows) on the periosteal surface of the calvaria was quantified on the right (*n* = 5). Scale bar, 100 μm. B, calvaria bone. **d**,**e**, Femoral bone defects were created in 8-week-old female C57BL/6 mice as described in the ‘Materials and methods’ section. Representative micro-CT images of the control, AQEE30 (3 μg), and PEG_2_-AQEE30 (3 μg) groups on day 10 postinjury are shown (**d**). The red circles indicate the hole where the injury occurred (day 0). The results of the quantitative analysis are shown (**e**) (*n* = 7). BMD, bone mineral density. The data represent the mean ± s.e.m. *P < 0.05, **P < 0.01, ***P < 0.001 versus the control.

We injected AQEE30 and PEG_2_-AQEE30 into the right side of the calvarial bone (Fig. 8a). H&E staining revealed that, compared with no PEG_2_-AQEE30 treatment, PEG_2_-AQEE30 treatment increased the total and new bone areas (Fig. 8b). Furthermore, compared with PBS treatment, PEG_2_-AQEE30 treatment increased calvaria thickness, total bone area and new bone area (Fig. 8b). In addition, compared with the control, PEG_2_-AQEE30 increased the number of osteocalcin-positive cells per bone surface (Fig. 8c). Furthermore, we investigated the effects of AQEE30 and PEG_2_-AQEE30 in a mouse femoral defect model (Fig. 8d,e). Compared with the control and AQEE30, PEG_2_-AQEE30 significantly decreased the bone defect area and increased the bone mass. These findings indicate that mini-PEGylated AQEE30 increases the number of bone-forming osteoblasts and bone formation in vivo.

## Discussion

This study was conducted to identify a novel factor that regulates bone formation, especially in cortical bone, from MK secretions. We identified VGF in fractions with the most significant difference in the ability to stimulate osteoblast viability using a proteomics approach. We demonstrated that AQEE30, a VGF-derived peptide, stimulates osteoblast proliferation and bone formation in vitro. Mice with *Vgf* deletion presented lower cortical bone mass and bone formation. Cellular studies revealed that cAMP–PKA signaling was essential for the action of AQEE30 in osteoblasts and that C3AR1 was a putative AQEE30 receptor. Injecting mini-PEGylated AQEE30 into calvaria increased the number of osteocalcin-positive cells and new bone formation. We identified a novel factor in MK secretions that acts on bones, and we demonstrated that AQEE30 modulates bone metabolism.

The strength of the present study is identifying the a MK-secreted factor that affects bone metabolism using an untargeted approach. VGF acts mainly through various active peptides after proteolytic cleavage in diverse cells and tissues^16^. VGF-derived peptides have various regulatory functions in neuronal activity, including neurogenesis, neurite outgrowth, neuronal survival and suppression of neuronal cell death^37–40^. VGF-derived peptides, including AQEE11, AQEE30, HHPD41 and LQEQ19, stimulated sympathetic outflow^41,42^. TLQP62 regulated hippocampal memory formation^43^. TLQP21 regulated various functions. For example, TLQP21 promoted the survival of cerebellar granule cells and prevented their apoptosis^44^. TLQP21 upregulated energy expenditure and blocked diet-induced obesity^45^. AQEE30 prevented cell death and aggregation of mutant huntingtin in STHdhQ111 cells^40^ and inhibited the loss of retinal ganglion cells while promoting neurite outgrowth of retinal ganglion cells in vitro^38^. However, to our knowledge, the roles of VGF and VGF-derived peptides in bone metabolism have not been reported.

Importantly, cortical bone mass and size were decreased in both male and female *Vgf*^−/−^ mice. Micro-CT analyses additionally revealed that the periosteal, but not the endosteal, perimeter was shorter in *Vgf*^−/−^ mice compared with their WT littermates. Thus, the cross-sectional size of the bone tissue, marked by Tt.Ar, was reduced. This finding is clinically important because cortical bone mass and size are critical for solid resistance to nonvertebral fracture^46^, suggesting that VGF could be an ideal therapeutic target to reduce nonvertebral fracture risk.

Unlike cortical bones, there were two inconsistencies in trabecular bones. First, the cortical bone mass decreased in male *Vgf*^−/−^ mice, whereas the trabecular bone parameters were not impaired on micro-CT. Although different mechanisms may regulate the two skeletal compartments^47–49^, the exact mechanism by which VGF deficiency affects the two compartments differently is unclear. The bone formation rate was not determined in trabecular bone because the labeling should be performed differently from that in cortical bone. However, histological findings revealed that the number of osteoblasts was decreased not only in the cortical bones but also in the trabecular bones of *Vgf*^−/−^ mice. In addition, *Vgf* was rarely expressed in skeletal stem progenitor cells in the periosteum and bone marrow (Supplementary Fig. 6a), and its receptors were expressed similarly between the two types of cells (Supplementary Fig. 6b,c). Therefore, the effect of *Vgf* deficiency on bone formation may be similar between the two compartments. However, we observed that periostin was expressed at lower levels in the periosteum of *Vgf*^−/−^ mice than in that of their WT littermates. It is well known that periostin, which is predominantly present in the periosteum^24^, stimulates bone formation by promoting the differentiation, migration and proliferation of osteoblasts^24^. This suggests that VGF may make the bone microenvironment more osteogenic at the periosteal surface. In addition, Vgf may influence other osteocyte-associated factors in addition to periostin because bone formation was observed at both the endocortical and periosteal surfaces of *Vgf*^−/−^ mice. In fact, we observed that the number of live osteocytes decreased in the cortical bones of *Vgf*^−/−^ mice. In addition, we cannot exclude the possibility that the discrepancy between cortical and trabecular bones may be due to delayed bone development because the *Vgf*^−/−^ mice were smaller than their WT littermates.

Second, we observed that cortical bone mass decreased in female *Vgf*^−/−^ mice, similar to that in male *Vgf*^−/−^ mice, but trabecular bone mass increased in *Vgf*^−/−^ female mice; this sex specificity was observed in trabecular bone but not in cortical bone. This finding indicates that there is no sex specificity in cortical bone in relation to the effects of VGF deficiency, but there is sex specificity in trabecular bone. The sex specificity of the effects of VGF deficiency on trabecular bone may be due to bone resorption; sex hormones may cause sex specificity because they strongly influence bone resorption. However, further studies on the precise mechanism by which *Vgf* deficiency affects trabecular bone resorption and formation in male and female mice are needed.

We previously reported that MK-CM stimulated osteoblastic proliferation, resulting in the stimulation of bone formation, but inhibited osteoblast differentiation^13^. AQEE30 increased osteoblastic bone formation by stimulating both proliferation and differentiation. Therefore, AQEE30 alone cannot mediate all the effects of MK secretions on osteoblasts. However, if it is limited to osteoblastic proliferation, AQEE30 is probably one of the most critical factors of MK secretions in osteoblastic proliferation based on the following evidence. First, AQEE30 levels were higher in CM from MK-like cells than in that from undifferentiated cells. Second, AQEE30 significantly stimulated the proliferation and viability of osteoblasts in a dose-dependent manner. Finally, CM obtained from VGF-depleted MK significantly inhibited the viability of MC3T3-E1 cells.

In the present study, PEG_2_-AQEE30, but not AQEE30, significantly increased calvarial bone formation by increasing the number of bone-forming cells in vivo. This result could be due to the increased in vivo stability of AQEE30 by PEGylation, but it could also be due to the increased activity of AQEE30 itself by PEGylation. This occurred because our cellular experiments revealed that PEG_2_-AQEE30 more strongly enhanced the binding to C3AR1, cAMP secretion and mineralization activities of osteoblasts than did AQEE30. Although the exact mechanism is unknown, it has been reported that PEGylation can regulate cell adhesion and growth^50,51^ and specifically promote the proliferation of human osteoblasts in vitro.

Previous studies have reported that the cAMP‒PKA pathway is crucial for inducing osteoblast differentiation and in vivo bone formation^28,52^. Furthermore, primary calvaria osteoblasts express the C3a receptor and regulate its expression during osteoblast differentiation through the C3a receptor^53^. Moreover, in this study, AQEE30 stimulated cAMP‒PKA signaling through interaction with the C3a receptor.

TLQP21, the most studied peptide among VGF-derived peptides, has diverse effects on various cells and organs, such as stimulating energy expenditure and metabolism, increasing glucose-induced insulin secretion, mediating stress responses and lipolysis, and regulating gastrointestinal functions^45,54–57^. Furthermore, TLQP21 acts as a ligand for C3AR1 (ref. ^58^). However, unexpectedly, TLQP21 did not affect the proliferation or differentiation of osteoblasts in the present study. In addition, treatment with TLQP21 did not change the intracellular cAMP level in MC3T3-E1 cells (data not shown), which is consistent with the findings of a previous study^59^. Although we did not demonstrate a difference in the mechanisms of action between AQEE30 and TLQP21 in osteoblasts, this may be because their coreceptors may differ. TLQP21 has at least two coreceptors, including the globular heads of the C1q receptor and heat shock protein family A member 8 (ref. ^60^), whereas the coreceptors for AQEE30 are unknown.

In summary, VGF secreted from MK-CM functions as an essential regulator of osteoblastic bone formation, especially in cortical bones. We identified AQEE30 as the main VGF-derived peptide that plays a role in activating cAMP‒PKA signaling via the C3AR1 receptor in osteoblasts (Supplementary Fig. 7). The results of this study provide a molecular basis for developing a therapeutic agent to reduce nonvertebral fracture risk.

## Supplementary information

Supplementary Information
