# Supplementary material for: VGF and the VGF-derived peptide AQEE30 stimulate osteoblastic bone formation through the C3a receptor
Source: Exp Mol Med. 2025 Mar 13;57(3):637–51. doi: 10.1038/s12276-025-01419-y (PMC11958639; doi:10.1038/s12276-025-01419-y)
Supplement: Supplementary file 1 — Supplementary Information [file 12276_2025_1419_MOESM1_ESM.pdf]

Supplementary Information

VGF and the VGF-derived peptide AQEE30 stimulate osteoblastic bone formation through the C3a receptor

Sung-Ah Moon, Jin-Man Kim, Young-Sun Lee, Han Jin Cho, Young Jin Choi, Jong Hyuk Yoon, Dayea Kim, Xiangguo Che, Xian Jin, In-Jeoung Baek, Seung Hun Lee, Je-Young Choi, and Jung-Min Koh

Supplementary Materials and Methods

Collection of Wnt3a CM

L929 cells stably overexpressing Wnt3a were cultured in DMEM supplemented with 10% FBS. After the cells reached 90% confluence, the medium was replaced with DMEM containing 2% FBS, and the cells were further incubated for 2 days. The supernatants were collected, filtered through a 0.45-μm filter, and stored at –80°C until further use.

Reporter gene assay

MC3T3-E1 cells (2×10^5 cells) were transfected with 100 ng superTOP-Flash, a Wnt signalling reporter, in 24-well plates using a Lipofectamine 2000 transfection reagent (Invitrogen), and treated with Wnt3a CM (1/10 dilution) in the presence or absence of 5 μM AQEE30 for 48 h. For the Runx2 reporter, the cells (2×10^5 cells) were transfected with 6×OSE2-luc Runx2 reporter constructs (100 ng), and treated with 1 μM and 5 μM AQEE30 for 48 h. Cell lysates were lysed in reporter lysis buffer and assayed with a Dual Luciferase kit (Promega, Madison, WI, USA). Luciferase activity was measured using a Luminometer (Centro LB 960; Berthold Technologies, Bad Wildbad, Germany).

VGF-derived peptides and antibodies

The VGF-derived peptides used in this study include NERP1 (#076-89), NERP2 (#076-91), AQEE19 (#007-73), AQEE30 (#007-70), TLQP21 (#003-90), and TLQP62 (#007-82) from Phoenix Pharmaceuticals (Burlingame, CA, USA). Mouse AQEE30 and H2N-8-amino-3,6-dioxaoctanoic acid (mini-PEG_2)-conjugated AQEE30 (PEG_2-AQEE30) were synthesized from Peptron Inc. (Daejeon, Korea). We used the following antibodies; Periostin (19899-1-AP) antibody from Proteintech (Rosemont, IL, USA); alkaline phosphatase (ALP, NBP3-15273) antibody from Novus Biologicals (Centennial, CO, USA); VGF (ab69989) antibody from Abcam (Cambridge, UK); p21 (sc-6246) antibody from Santacruz Biotechnology (Dallas, TX, USA); His (12698), pRb (9308), and Rb (9309)

antibodies from Cell signaling (MA, USA); α-tubulin (T9026) and actin (A3854) antibodies from Sigma-Aldrich; osteocalcin (M173) antibody from Takara (Tokyo, Japan); and C3AR1 (orb101135) antibody from Biorbyt (Cambridge, UK).

Isolation of mouse periosteal skeletal stem cells (PSSC) and bone marrow skeletal stem cells (BMSC)

After careful removal of the muscles, femurs and tibias were incubated with PBS containing 1% FBS on ice for 30 min to loosen the periosteum from the underlying bone surface. Periosteum was detached from the bones using scalpel and forceps and placed on a cell strainer. The collected periosteal tissues washed with cold PBS and incubated with PBS containing collagenase (1 mg/ml, Gibco) and dispase (2 mg/ml, Sigma-Aldrich) at 37°C for 60 min. The dissociated cells were washed with PBS, filtered through a 40-μm nylon strainer, and resuspended in MEMα containing 10% FBS. For isolation of BMSC, BM cells were collected from the dissected femurs and tibias using a syringe with a 26-gauge needle. The BM cells were incubated with collagenase (1 mg/ml) and dispase (2 mg/ml) in PBS at 37°C for 60 min. The dissociated BM cells were washed with PBS, filtered using a 40-μm strainer. The cells were resuspended in MEMα with 10% FBS.

Colony forming unit assay

PSSC (35 ~ 40 cells/well) were seeded on a 6-well plates in MEMα medium with 20% FBS. After 24 h, the medium was treated with $Vgf^{+/+}$ and $Vgf^{-/-}$ MK-CM and cultured for 14 days. The cells were washed, fixed with 4% paraformaldehyde, and stained with 1% crystal violet staining solution according to the manufacturer's protocol.

Chondrogenic differentiation assay

PSSC (2×10^{5}) were plated in a 12-well plate for 2 days. The cells were cultured with MK-CM from

Vgf$^{+/+}$ *and Vgf*$^{-/-}$ mice in the presence of chondrogenic differentiation reagent (Gibco). The medium was changed every 2-3 days for 21 days and the cells were stained with Alcian blue solution (Sigma-Aldrich) according to the manufacturer's protocol. The stained Alcian blue dye was dissolved with 6M Guanidine-HCl solution (Sigma-Aldrich) and measured using a microplate reader at absorbance 620 nm.

ALP immunhistochemistry and osteocyte counting

Femurs were fixed in 4% paraformaldehyde, decalcified, and sectioned with a cryostat. The 5-μm-thick paraffin-embedded bone sections were deparaffinized, rehydrated, and subjected to heat-mediated antigen retrieval in citrate buffer at pH 6.0. The slides were incubated with an anti-ALP antibody (1/100 dilution; Novus Biologicals) at 4°C overnight. The slides were incubated with the Dako Envision+ System-horseradish peroxidase-labeled polymer secondary antibody system (Dako K4003) for 60 min at room temperature. The slides were stained with the Dako Liquid DAB+ substrate chromogen system (Dako K3467) according to the manufacturer's instructions, and were counter-stained with hematoxylin. Images were photographed using an Olympus BX53 microscope (Olympus Corporation). For counting of osteocytes, the bone section slides were stained with H&E. The slides were photographed and measured with IMT i-Solution Lite software (version 26.1; IMT i-Solution Inc., BC, Canada). Osteocytes were classified histologically into alive, dying, and dead cells as described a previous study.[1] Alive cells were characterized by a whole nucleus with almost no empty space in the lacuna. Dying cells were identified as cells with a fragmented nucleus in the lacuna. Dead cells were identified as having no cell and nucleus in the lacuna. Quantitative analysis was calculated by dividing the number of counted cells/lacunae by the area of cortical bone or total number of cells/lacunae.

Supplementary Reference

1. McKenzie J, Smith C, Karuppaiah K, Langberg J, Silva MJ, Ornitz DM. Osteocyte Death and

Bone Overgrowth in Mice Lacking Fibroblast Growth Factor Receptors 1 and 2 in Mature Osteoblasts and Osteocytes. *J Bone Miner Res* 2019; **34:** 1660-1675.

Supplementary Table 1. Sequences of siRNAs.

Primers	Forward (5'-3')
Human *VGF*	CGGCGGCAGCAGAGACGGAAA
Scrambled (*VGF*)	GCGAACGGCGACGGAGGACAA
Mouse *C3AR1*	CAGCGGGTTTCCTGTTGAAGA
Scrambled (*C3AR1*)	GTTGTTAGCGTGGACATCCAG

Supplementary Table 2. Primer sequences for qRT-PCR.

Primers	Forward (5'-3')	Reverse (5'-3')
Human *VGF*	AGCCTCCTCCTCTCAGCTCT	GAAAAGCTCTCCCTCGTCCT
Human *HMGB1*	GCGAAGAAACTGGGAGAGATGT G	GCATCAGGCTTTCCTTTAGCTCG
Human *HDGF*	AGAGGCTACCAAGGAAGATGC	GGCTCCTCTTGAAACATTGG
Human *18S*	GTAACCCGTTGAACCCCATT	CCATCCAATCGGTAGTAGCG
Mouse *Vgf*	ATACTCCAGCCACGGAACAG	GGCAACGTGAAGGTTTTCAT
Mouse *C3ar1*	TGACAGGTCAGCTCCTTCCT	CATTAGGAGGCTTTCCACCA
Mouse *gC1qR*	GGCCTTCGTTGAATTCTTGA	TCCGCAAGGAAATCCATTAG
Mouse *18S*	CTCAACACGGGAAACCTCAC	CGCTCCACCAACTAAGAACG

Supplementary Table 3. Proteins commonly identified in two fractions of conditioned media collected from differentiated megakaryocyte-like cells.

Accession Number	Symbol	Score	Molecular weight (kDa)	Fraction #51		Fraction #51	
				Coverage	Matched peptides	Coverage	Matched peptides
O15240	VGF	37.9	67.2	11.7	8	9.1	6
P09429	HMGB1	24.2	24.9	7.9	6	9.3	3
P51858	HDGF	10.5	26.8	18.8	3	7.1	1
P07737	PFN1	2.5	15.0	10.0	1	11.4	2
P35637	FUS	16.7	53.4	6.3	4	12.4	4
P42167	LAP2	13.0	50.6	5.5	4	5.7	1
P62888	RPL30	6.0	12.8	7.0	1	7.0	1
Q13185	CBX3	6.8	20.8	7.7	2	7.7	1
Q6ZSJ8	C1orf122	2.9	11.5	10.0	1	10.0	1
Q96KK5	H2AC12	5.4	13.9	14.8	1	37.5	3
Q9Y237	PIN4	17.3	13.8	10.7	4	10.7	1
Q9Y2V2	CARH2P1	5.9	15.9	18.4	2	10.9	1

The three proteins in the grey background may be secreted extracellularly.

Supplementary Table 4. Identification of VGF in conditioned media collected from differentiated megakaryocyte-like cells.

1	MKALRLSASA	LFCLLLINGL	GAAPPGRPEA	QPPPLSSEHK	EPVAGDAVPG
51	PKDGSAPEVR	GARNSEPQDE	GELFQGVDPR	ALAAVLLQAL	DRPASPPAPS
101	GSQQGPEEEA	AEALLTETVR	SQTHSLPAPE	SPEPAAPPRP	QTPENGPEAS
151	DPSEELEALA	SLLQELRDFS	PSSAKRQQET	AAAETETRTH	TLTRVNLESP
201	GPERVWRASW	GEFQARVPER	APLPPPAPSQ	FQARMPDSGP	LPETHKFGEG
251	VSSPKTHLGE	ALAPLSKAYQ	GVAAPFPKAR	RPESALLGGS	EAGERLLQQG
301	LAQVEAGRRQ	AEATRQAAAQ	EERLADLASD	LLLQYLLQGG	ARQRGLGGRG
351	LQEAAEERES	AREEEEAEQE	RRGGEERVGE	EDEEAAEAEA	EAEEAERARQ
401	NALLFAEEED	GEAGAEDKRS	QEETPGHRRK	EAEGTEEGGE	EEDDEEMDPQ
451	TIDSLIELST	KLHLPADDVV	SIIEEVEEKR	KRKKNAPPEP	VPPPRAAPAP
501	THVRSPQPPP	PAPAPARDEL	PDWNEVLPPW	DREEDEVYPP	GPYHPFPNYI
551	RPRTLQPPSA	LRRRHYHHAL	PPSRHYPGRE	AQARRAQEEA	EAEERRLQEQ
601	EELENYIEHV	LLRRP			

Among the sequences of the VGF protein, those of the peptides identified by LC-MS/MS are highlighted in red-colored text.

Supplementary Figure 1

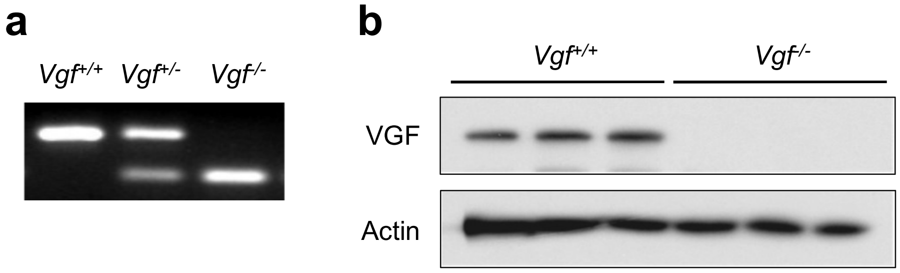

Supplementary Fig. 1. Genotyping and VGF expression in male *Vgf*$^{-/-}$ mice. a Genotyping PCR analysis of *Vgf*$^{+/+}$, *Vgf*$^{+/-}$, and *Vgf*$^{-/-}$ alleles. The bands corresponding to the *Vgf*$^{+/+}$ and *Vgf*$^{-/-}$ allele are 125 bp and 64 bp, respectively. **b** Total proteins were extracted from calvaria bone of male *Vgf*$^{+/+}$ and *Vgf*$^{-/-}$ mice, and western blot for VGF was performed.

Supplementary Figure 2

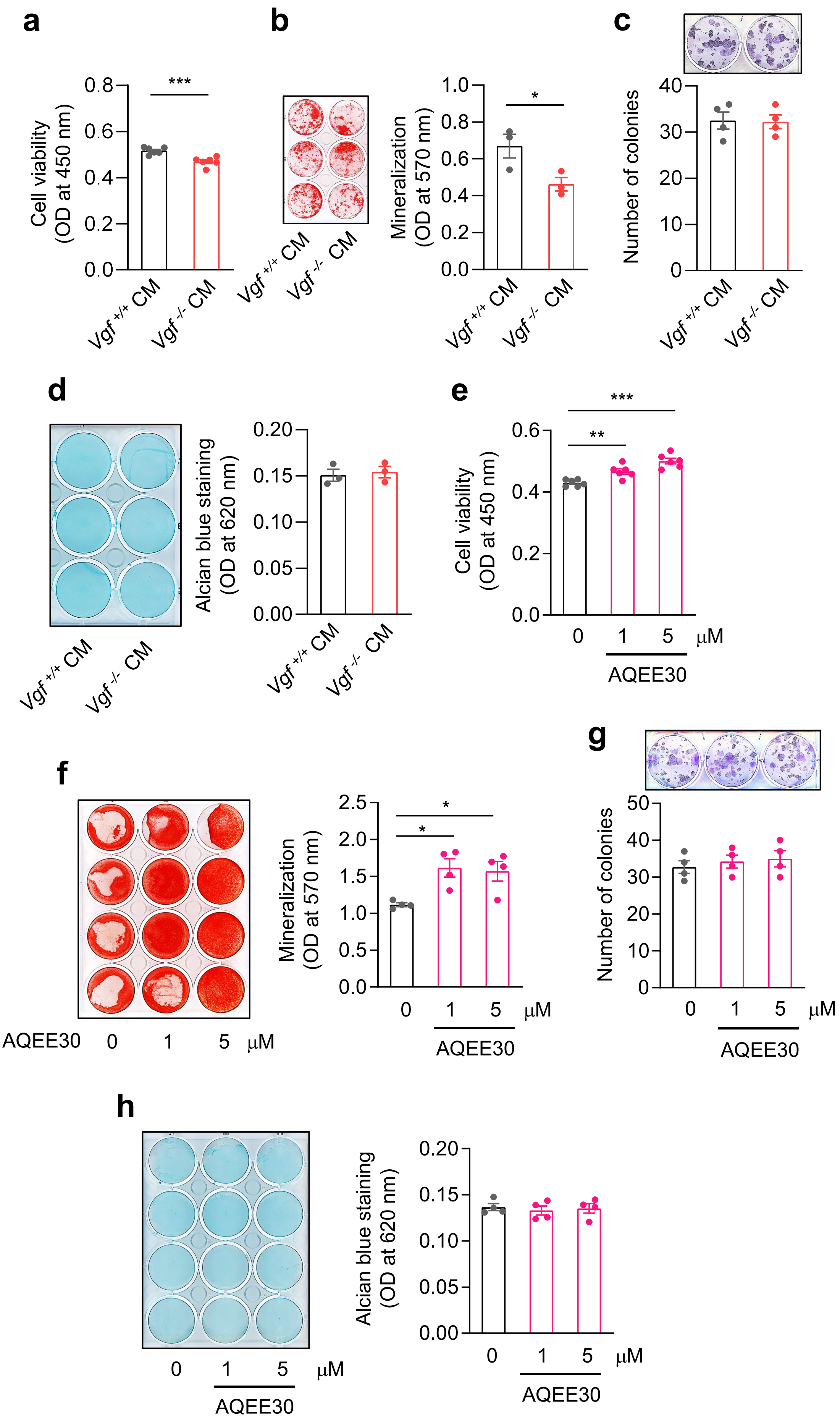

Supplementary Fig. 2. Effects of MK CM and AQEE30 on periosteal skeletal stem cells. a, **b** Murine fetal liver cells isolated from *Vgf*$^{-/-}$ mice and their wild-type littermates were differentiated into mature megakaryocytes (MK) by thrombopoietin treatment. Cell viability (**a**, $n = 6$) and mineralization capacity (**b**, $n = 3$) of periosteal skeletal stem cells (PSSC) after the treatment with conditioned media (CM) of mature MK were measured. Mineralization were stained with Alizarin red S (left), and measured at 570 nm (right). **c** PSSC were treated with mature MK CM and the number of colony formation was counted after 14 days. Colonies consisting of more than 50 cells were counted (lower, $n = 4$). **d** Chondrogenic differentiation assay was determined by Alcian blue staining (left) and measured at 620 nm (right) ($n = 3$). **e** PSSC were treated with AQEE30 for 48 h, and cell viability was determined ($n = 6$). **f** Bone nodule formation assays in PSSC was carried out as in (**b**) after treatment with the indicated doses of AQEE30 ($n = 4$). **g** PSSC were treated with AQEE30 and the number of colony formation was counted after 14 days ($n = 4$). **h** Chondrogenic differentiation assay was determined by Alcian blue staining (left) and measured at 620 nm (right) ($n = 4$). Data represent mean ± SEM. *$p < 0.05$, **$p < 0.01$, and ***$p < 0.001$ vs. control groups.

Supplementary Figure 3

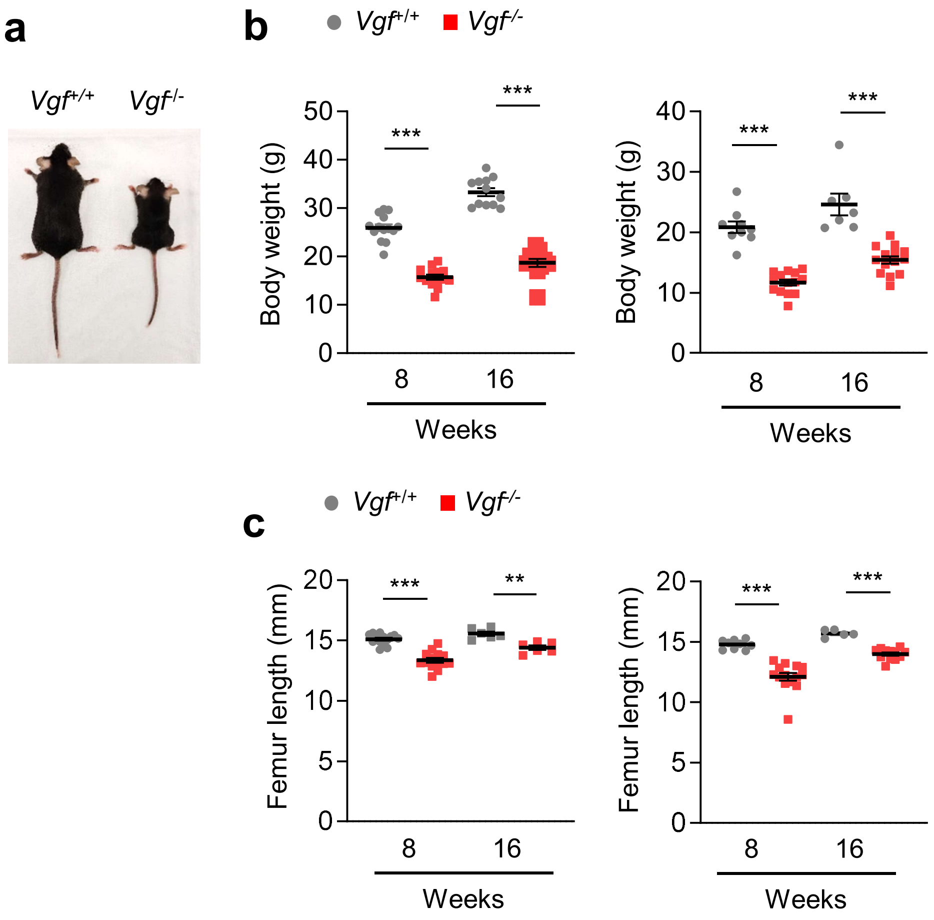

Supplementary Fig. 3. Postnatal growth in both male and female *Vgf*$^{-/-}$ mice. **a–c** Gross appearance (**a**), body weight (**b**), and femur length (**c**) of male (left) and female (right) *Vgf*$^{-/-}$ mice and their wild-type littermates at 8 and 16 weeks of age. Data represent mean ± SEM (**b**, 14 *Vgf*$^{+/+}$ and 14 *Vgf*$^{-/-}$ mice in 8-week-old male, 12 *Vgf*$^{+/+}$ and 11 *Vgf*$^{-/-}$ mice in 16-week-old male, 9 *Vgf*$^{+/+}$ and 16 *Vgf*$^{-/-}$ mice in 8-week-old female, and 7 *Vgf*$^{+/+}$ and 14 *Vgf*$^{-/-}$ mice in 8-week-old female; **c**, 14 *Vgf*$^{+/+}$ and 14 *Vgf*$^{-/-}$ mice in 8-week-old male, 6 *Vgf*$^{+/+}$ and 6 *Vgf*$^{-/-}$ mice in 16-week-old male, 9 *Vgf*$^{+/+}$ and 16 *Vgf*$^{-/-}$ mice in 8-week-old female, and 5 *Vgf*$^{+/+}$ and 12 *Vgf*$^{-/-}$ mice in 16-week-old female). **$p < 0.01$ and ***$p < 0.001$ between the groups.

Supplementary Figure 4

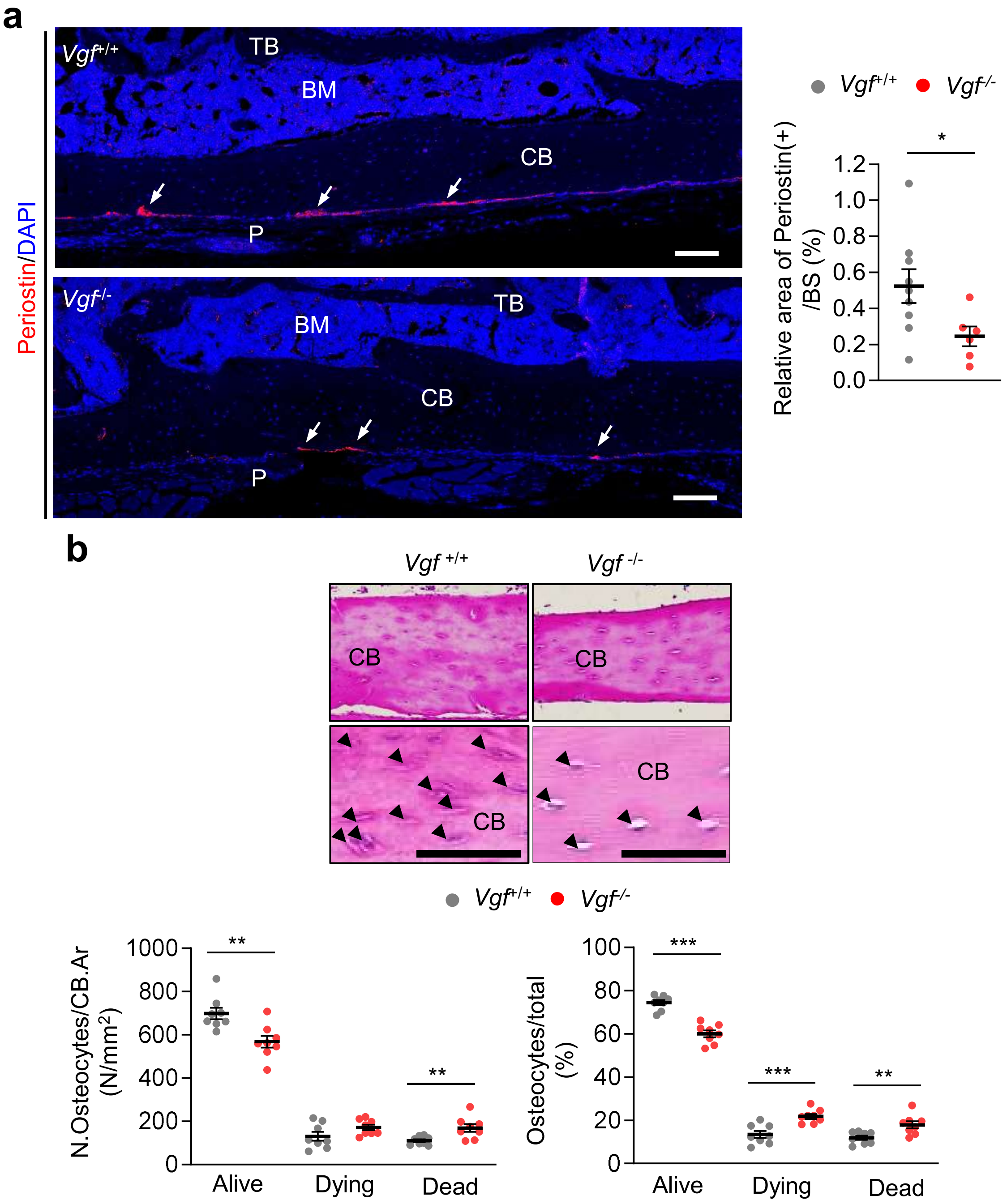

Supplementary Fig. 4. Decreased periostin expression and osteocyte numbers in the cortical bones of ***Vgf*****$^{-/-}$ mice. a** Periostin areas (white arrows) of 16-week-old male *Vgf*$^{-/-}$ mice (n = 6) and their wild-type littermates (n = 9). Representative images are shown on the left, and their quantitative result is shown on the right. Scale bar, 100 µm. White arrows indicate periostin-positive areas. **b** Representative hematoxylin and eosin staining images of femurs of 16-week-old male *Vgf*$^{-/-}$ mice (n = 8) and their *Vgf*$^{+/+}$ littermates (n = 8) are shown on the upper. Osteocyte number (N.Osteocytes) per cortical bone areas (CB.Ar) (left/bottom) and total osteocytes (right/bottom) are shown. Scale bar, 50 µm. Arrow heads indicate the osteocytes. BM, bone marrow; BS, bone surface; CB, cortical bone; TB, trabecular bone; and P, periosteum. Data represent mean ± SEM. $*p < 0.05$, $**p < 0.01$, and $***p < 0.001$ between the groups.

Supplementary Figure 5

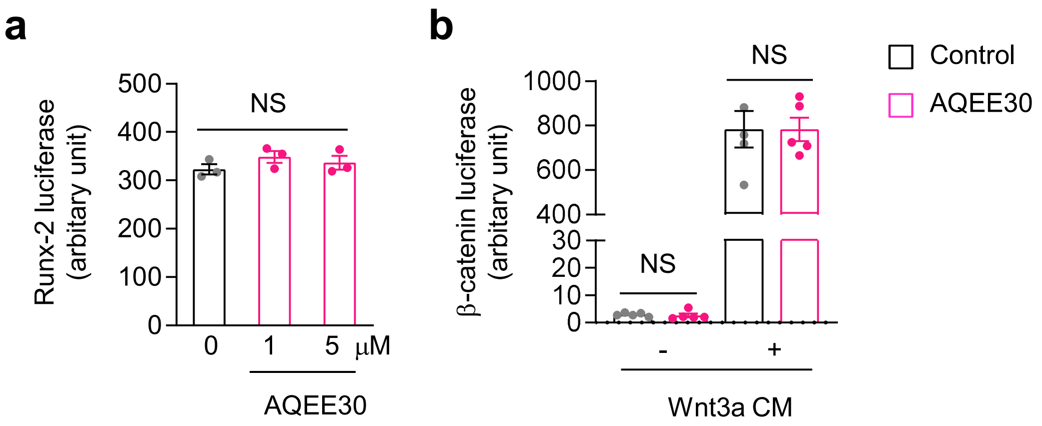

Supplementary Fig. 5. Effects of AQEE30 on Runx2 and β-catenin activities. a MC3T3-E1 cells were treated with 1 μM and 5 μM AQEE30 for 48 h, and Runx2 transcriptional activity was measured using a Runx2 luciferase assay. Data represent mean ± SEM (n = 3). **b** MC3T3-E1 cells were treated with 5 μM AQEE30 in the presence or absence of Wnt3a CM for 48 h, and the β-catenin luciferase assay was performed. Data represent mean ± SEM (n = 5). NS, not significant.

Supplementary Figure 6

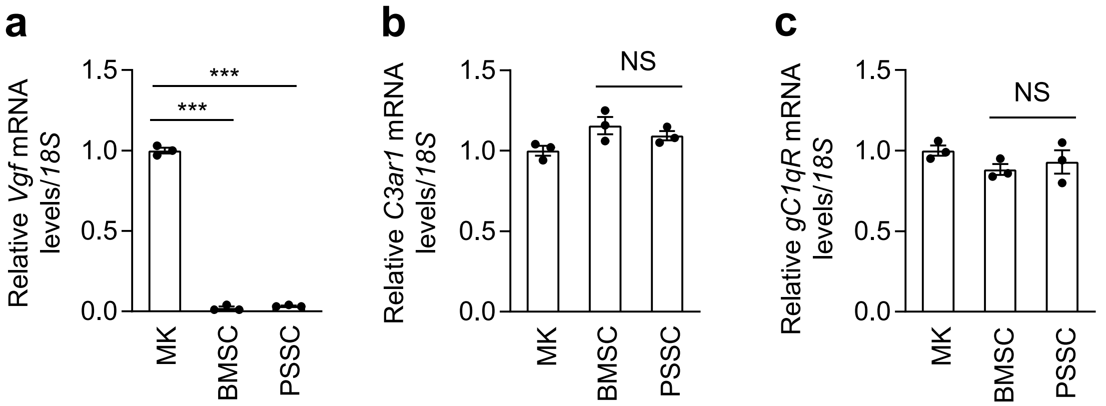

Supplementary Fig. 6. Expressions of *Vgf* and its receptors in MK and skeletal stem cells from periosteum and bone marrow. a-c, Bone marrow skeletal stem cells (BMSC), periosteal skeletal stem cells (PSSC), and megakaryocytes (MK) were isolated from mice . Relative expression of mRNA was determined by real-time PCR. Data represent mean ± SEM ($n = 3$). ***$p < 0.001$ between the groups. NS, not significant.

Supplementary Figure 7

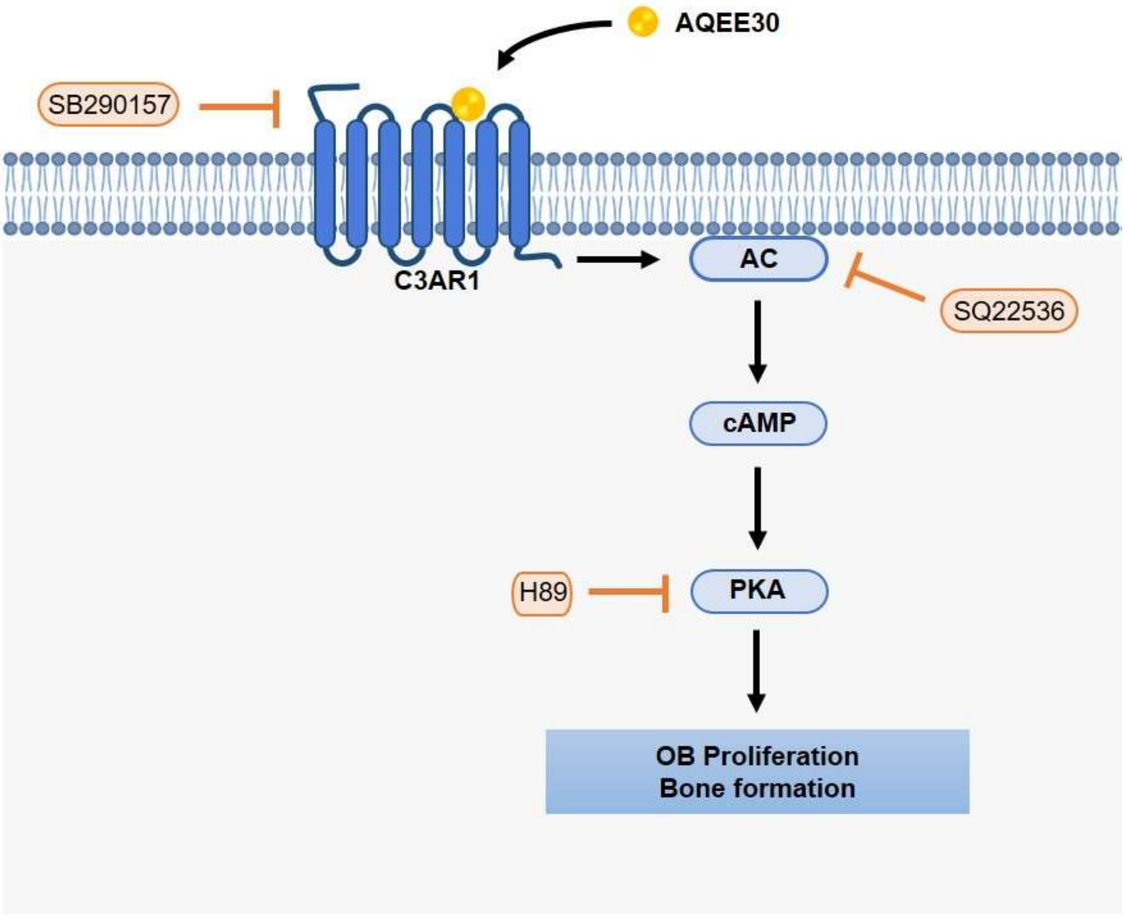

Supplementary Fig. 7. A proposed model of AQEE30's roles on osteoblastic proliferation and bone formation. AQEE30 activates the C3AR1 and consequently triggers the AC-cAMP-PKA signalling pathway, which in turn promotes osteoblastic proliferation and bone formation. C3AR1, complement C3a receptor-1; AC, adenylyl cyclase; cAMP, cyclic adenosine monophosphate; OB, osteoblast; PKA, protein kinase A.
